# CRISPR/dCas9 DNA methylation editing is heritable during human hematopoiesis and shapes immune progeny

**DOI:** 10.1073/pnas.2300224120

**Published:** 2023-08-14

**Authors:** Emily A. Saunderson, Hector Huerga Encabo, Julie Devis, Kevin Rouault-Pierre, Marion Piganeau, Christopher G. Bell, John G. Gribben, Dominique Bonnet, Gabriella Ficz

**Affiliations:** ^a^Centre for Haemato-Oncology, Barts Cancer Institute, John Vane Science Centre, Charterhouse Square, Queen Mary University of London, London EC1M 6BQ, United Kingdom; ^b^Haematopoietic Stem Cell Laboratory, Francis Crick Institute, London NW1 1AT, United Kingdom; ^c^Group of Computational Biology and Bioinformatics, de Duve Institute, Université Catholique de Louvain, Brussels 1200, Belgium; ^d^William Harvey Research Institute, Barts and the London Faculty of Medicine and Dentistry, Queen Mary University of London, London EC1M 6BQ, United Kingdom

**Keywords:** epigenetic editing, CRISPR, hematopoiesis

## Abstract

Aging is associated with an abnormal increase in DNA methylation (DNAm) in human gene promoters, including in bone marrow stem cells. DNAm patterns are further perturbed in hematological malignancies such as acute myeloid leukemia but the physiological significance of such epigenetic changes is unknown. Using epigenetic editing of human stem/progenitor cells (HSPCs), we show that p15 methylation affects hematopoiesis in vivo. We edited the *CDKN2B* (p15) promoter and *ARF* (p14) using dCas9-3A3L and observed DNAm spreading beyond the gRNA location. We find that despite a transient delivery system, DNAm is maintained during myeloid differentiation in vitro, and hypermethylation of the p15 promoter reduces gene expression. In vivo, edited human HSPCs can engraft the bone marrow of mice and targeted DNAm is maintained in HSPCs long term. Moreover, epigenetic changes are conserved and inherited in both myeloid and lymphoid lineages. Although the proportion of myeloid (CD33^+^) and lymphoid (CD19^+^) cells is unaffected, monocyte (CD14^+^) populations decreased and granulocytes (CD66b^+^) increased in mice engrafted with p15 hypermethylated HSPCs. Monocytes derived from p15 hypermethylated HSPCs appear to be activated and show increased inflammatory transcriptional programs. We believe these findings have clinical relevance since we found p15 promoter methylation in the peripheral blood of patients with clonal hematopoiesis. Our study shows DNAm can be targeted and maintained in human HSPCs and demonstrated functional relevance of aberrant DNAm on the p15 locus. As such, other aging-associated aberrant DNAm may impact hematopoiesis in vivo.

## Significance

Understanding the contribution of epigenetic mechanisms to cancer initiation became paramount since we know that most healthy tissues carry mutations, but only a minority undergo malignant transformation. We developed methodologies which allow locus-specific epigenetic editing of the DNA sequence, using a dCas9-effector fusion in primary human hematopoietic stem and progenitor cells (HSPCs) to investigate the impact of cancer-specific DNA methylation (DNAm) changes on cell physiology. We uncover principles of DNAm maintenance and the impact on mature cells as HSPCs differentiate in vitro and in vivo.

The development of CRISPR/Cas9 for gene editing has revolutionized our ability to manipulate the genome (1–3). CRISPR/Cas9 has been further developed and adapted to enable precise control over genetic, epigenetic, and transcriptional processes (4–7). Different versions and combinations of CRISPR/dCas9 constructs, where Cas9 is catalytically inactive (dCas9), are fused to DNA methyltransferases (DNMTs) and histone modifiers to achieve epigenome editing. These tools can efficiently target one of the most well-studied epigenetic modifications, DNA methylation (DNAm), leading to addition of a methyl group to a cytosine forming 5-methylcytosine to virtually any desired location in the genome. We and others have previously demonstrated that targeting CRISPR/dCas9 DNMT to gene promoter CpG islands causes methylation spreading and subsequent gene repression (8–14). Although early studies hinted at the potential to use these tools to understand the causal roles of DNA hypermethylation in normal cell biology and disease, there have been several technical caveats. For example, some studies have shown significant off-target hypermethylation effects to the surrounding genome (15); additionally, targeted DNAm is often not maintained [with rare exceptions (8, 10)] in cell lines, and therefore the physiological impact of targeting DNAm has been difficult to determine.

Our previous work using primary human breast myoepithelial cells challenged these widely accepted limitations of DNAm targeting. Targeting DNAm to p16 (*CDKN2A*) using dCas9 3A3L [DNMT3A catalytic domain fused to portion of DNMT3L (9)] altered the biology of the primary cells, enabling them to proliferate in culture for at least 3 months longer than under normal conditions (10). We saw minimal off-target effects across the genome, suggesting that targeting biologically relevant genes in primary cells may increase the functional relevance of DNAm targeting while reducing off-target effects.

We wanted to expand our finding of seemingly permanent DNAm maintenance in primary breast cells, by developing a method for epigenetic editing in the hematopoietic system. Hematopoiesis is one of the most well-defined differentiation hierarchies, with established markers for cellular identity and is therefore an excellent model system to begin exploring DNAm maintenance after de novo targeting in hematopoietic stem and progenitor cells (HSPCs). Introducing a Cas9 nuclease into HSPCs, although challenging, has been demonstrated by others resulting in efficient multiplex editing of mutations in these cells (16, 17).

Gene editing in human HSPCs has been used to assess functionally the impact of mutations commonly found in acute myeloid leukemia (AML) to create useful disease models, but surprisingly no hits are sufficient to initiate overt leukemia in mice (16). This suggests additional disruption to cellular function is required to promote disease [e.g., additional mutational burden, changes to the inflammatory network intracellularly and/or from the microenvironment (18–20)]. In addition, it may be necessary for cells to acquire pathological epigenetic “hits” to alter the (epi)genetic landscape and promote immortalization and cancer. For example, aberrant p15 (*CDKN2B*) promoter hypermethylation has been found frequently [reportedly up to 80% of patients (21)] in myeloid dysplasia/neoplasm and AML (22–25), but whether p15 hypermethylation impacts cell biology or contributes to the development of disease is unknown due to a lack of functional experiments.

In this study, we have optimized the delivery system for dCas9 3A3L to multiplex DNAm editing in human CD34^+^ HSPCs from umbilical cord blood. From bulk and single-colony analyses, we show that in the absence of the editing tool DNAm is maintained at p14 and p15 as myeloid cells differentiate, with some CpGs remaining up to 99% methylated. We have combined epigenetic and genetic editing to demonstrate the feasibility of creating multiple aberrant hits within the same CD34^+^ cell. The edited human HSPCs can engraft murine bone marrow and recapitulate hematopoiesis with effects on differentiation in immune cell populations, which may lead to an inflammatory environment in vivo. p15 methylation is heritable in both myeloid and lymphoid lineages, further demonstrating the usability of this method for generating disease-relevant mouse models of human hematopoietic malignancy. Our study shows CRISPR/dCas9 DNAm editing in primary human HSPCs is possible and gives insights into the maintenance of de novo methylation during hematopoiesis in vitro and in vivo.

## Results

### Epigenetic Editing of Human HSPCs.

**Fig. 1.**
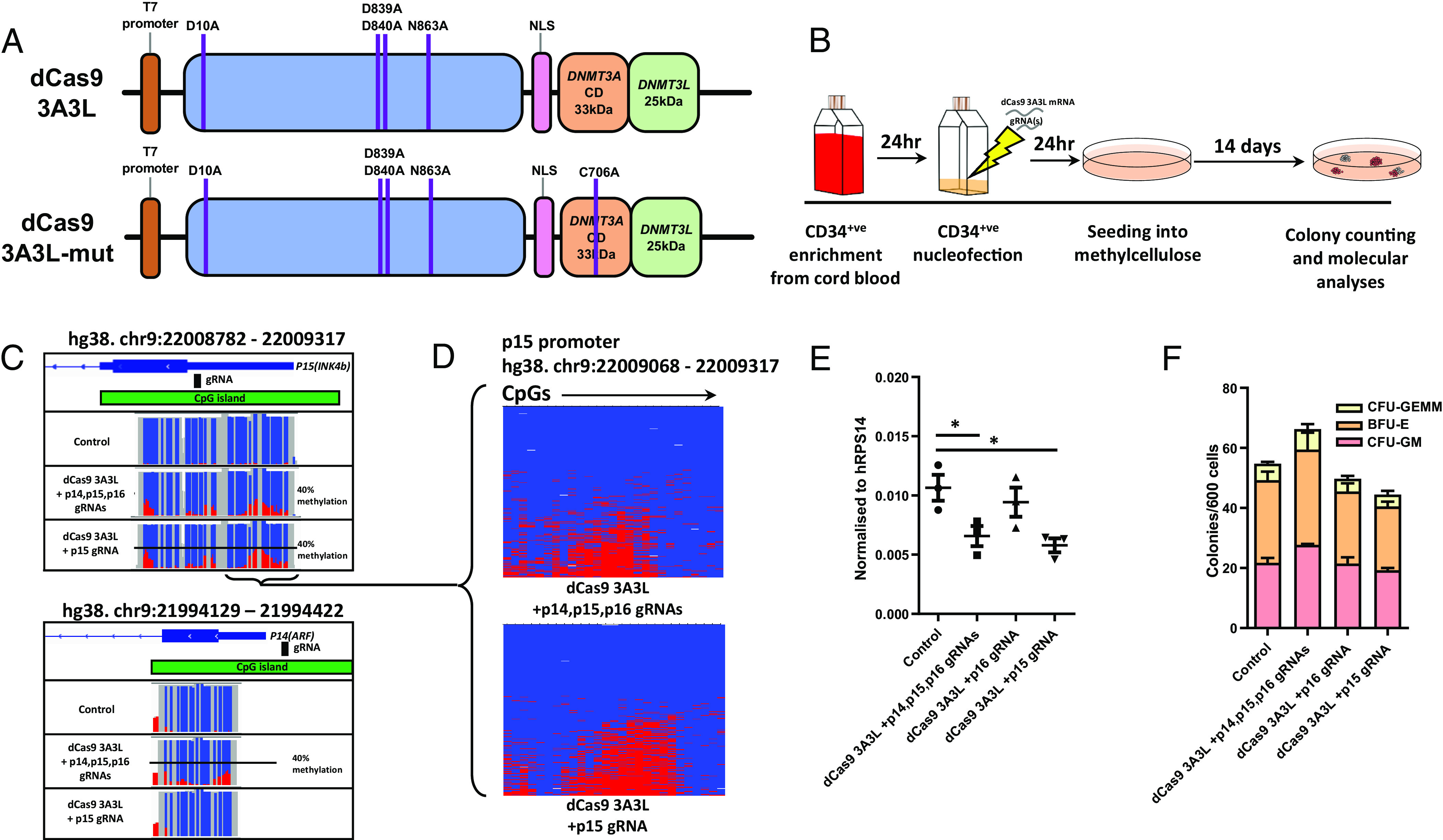
dCas9 3A3L targets DNA hypermethylation at p14 and p15 promoters in primary human HSPCs. (*A*) The dCas9 has amino acid substitutions resulting in catalytic inactivity and is fused to catalytic domains of DNMT3A, and a short sequence of its regulator DNMT3L. dCas9 3A3L-mut has no catalytic activity as it contains a C706A substitution in DNMT3A. (*B*) An outline of the experimental set up as described in the *Methods*. (*C*) Targeted bs-seq data from bulk colonies harvested after CFU visualized using the Integrative Genomics Viewer. Percentage DNAm data from p15 (*Top*) and p14 (*Bottom*) is displayed with fraction of unmethylated (blue) and hypermethylated (red) shown as vertical lines; each line represents data from an individual CpG. (*D*) Targeted bs-seq data visualized as individual DNA strands (rows) and CpGs (columns) at the p15 promoter after targeting DNAm using gRNAs to p14, p15 and p16 (*Upper*); and targeting p15 alone (*Lower*) followed by 14 d CFU assay and bulk colony harvest. (*E*) Gene expression of p15 in bulk colonies after methylation targeting and 14 d in a CFU assay. qPCR data are normalized to RPS14 expression (n = 3; one-way ANOVA with Dunnett’s post hoc test; *P < 0.05 compared to Control. (*F*) Number and type of colonies counted after methylation targeting and seeding 600 cells in methylcellulose before 14 d in the CFU assay (n = 3; Two-way ANOVA ns.). CFU-GEMM; colony-forming unit granulocyte, erythrocyte, monocyte, megakaryocyte. BFU-E; burst-forming unit-erythroid. CFU-GM; colony-forming unit granulocyte, monocyte.

To establish a method for targeting DNAm in HSPCs, we used the dCas9 3A3L and 3A3L-mut plasmids from our previous work (10), and others (9), and cloned a T7 promoter upstream of the dCas9 region (Fig. 1*A*). We then performed in vitro transcription and checked for mRNA product (*SI Appendix*, Fig. S1*A*). Human CD34^+^ cells were isolated from umbilical cord blood and cultured in serum-free conditions with cytokines Flt-L (fms-related tyrosine kinase 3 ligand), SCF (stem cell factor), and TPO (thrombopoietin) for 24 h (17) before nucleofection with dCas9 3A3L or 3A3L-mut mRNA and combinations of single guide RNAs (gRNAs) targeting p14 (*ARF*), p15 (*CDKN2B*) and p16 (*CDKN2A*) gene promoters (Fig. 1*B*). Nucleofection with a GFP mRNA showed there was a high efficiency of mRNA uptake in CD34^+^ cells (*SI Appendix*, Fig. S1*B*). After nucleofection, CD34^+^ cells were cultured with cytokines for a further 24 h before seeding into semisolid methylcellulose containing cytokines promoting myeloid differentiation in a colony-forming unit (CFU) assay. After 2 wk, colonies were counted and harvested for molecular analysis.

### DNAm Is Maintained during Myeloid Differentiation.

We pooled colonies from the CFU-assay and using targeted bisulfite sequencing (bs-seq) found targeted DNAm across the p15 promoter (a maximum of 43% methylation at individual CpGs) when using gRNAs targeting p15 alone and when using gRNAs to both p14 and p15 (39% maximum at p15; 35% maximum at p14; Fig. 1*C*). Interestingly, we found DNAm had spread across the p15 promoter (488 bp region measured) and p14 promoter (221 bp region measured), as has been shown previously (9, 10). Visualizing individual DNA strands at the p15 promoter showed that CpG methylation was across the same DNA strand and therefore within the same cell (Fig. 1*D*; one region of p15 promoter). Three independent replicates showed a significant increase in CpG methylation at the p14 and p15 promoters after nucleofection and the CFU assay (*SI Appendix*, Fig. S1*C*). In contrast to our previous work using these gRNAs (10), in HSPCs we did not detect targeted methylation at the p16 promoter after the CFU assay (*SI Appendix*, Fig. S1*D*). We checked publicly available chromatin immunoprecipitation data (26) from CD34^+^ cells compared to primary myoepithelial cells, which we used previously, but did not find any differences in H3K4me3, H3K9me3, or H3K27me3 at the p16 promoter (*SI Appendix*, Fig. S2). We reason there are other structural or chromatin signals that preclude hypermethylation of p16 in HSPCs.

We also cultured cells in serum-free conditions for 14 d with SCF, Flt3-L, and TPO after DNAm targeting and found similar levels of methylation compared to the CFU assay (*SI Appendix*, Fig. S3*A*), indicating that different cytokine cocktails and culture conditions did not affect methylation maintenance. Finally, we found that increasing the total picomols of dCas9 3A3L mRNA and gRNAs nucleofected resulted in more hypermethylation at the p15 promoter after the CFU assay (*SI Appendix*, Fig. S3*B*). We checked for successful epigenetic editing 2 d after nucleofecting CD34^+^ cells with dCas9 3A3L mRNA and gRNAs followed by culturing in serum-free conditions. We found that at 2 d postnucleofection a small percentage (<5%) of cells had evidence of DNAm at the p15 promoter when cells were nucleofected with a single gRNA targeting p15 (*SI Appendix*, Fig. S3*C*). We speculate that this is most likely because the differentiated and dead cells in the population are not successfully edited.

### Targeted DNAm Reduces p15 Expression, but Colony-Forming Capacity Is Unchanged.

We next checked whether targeted DNAm resulted in heritable gene repression following the CFU assay. We found that differentiated cells with hypermethylation at p15 consistently had lower expression of this gene (Fig. 1*E* and *SI Appendix*, Fig. S3*D*). Gene expression of p16 and p14 was not significantly different between groups (*SI Appendix*, Fig. S3*E*). This indicates that DNAm is maintained and the impact on gene expression persists at p15 as myeloid progenitor cells differentiate. We counted the total number of colonies formed to quantify whether targeting DNAm caused skewing of CD34^+^ cell differentiation, but there were no significant differences (Fig. 1*F*), suggesting that myeloid progenitor differentiation in the CFU assay is not affected by p14 and p15 promoter methylation.

### Off-Target DNAm Events Are Minimal to None.

We analyzed genome-wide DNAm after the CFU assay using the Illumina Infinium MethylationEPIC (850k) array. In this experiment, we included an additional control group of dCas9 3A3L and a nontargeting gRNA, as well as dCas9 3A3L-mut, as active dCas9 3A3L has been shown to cause off-target hypermethylation (15). We found no significant differentially methylated probes (DMPs) between the nontargeting vs. dCas9 3A3L-mut groups by implementing a strict Bonferroni correction, indicating no maintained off target DNAm events driven by the dCas9 3A3L alone. We found three significantly hypermethylated CpGs in dCas9 3A3L vs. 3A3L-mut (*SI Appendix*, Table S1) all residing within *CDKN2B* and the same significantly hypermethylated CpGs comparing dCas9 3A3L to the nontargeting control (*SI Appendix*, Table S2). This showed there were no statistically significant off-target methylation effects maintained in the colonies.

### gRNA Location Is Critical for Successful DNAm Targeting at p15.

**Fig. 2.**
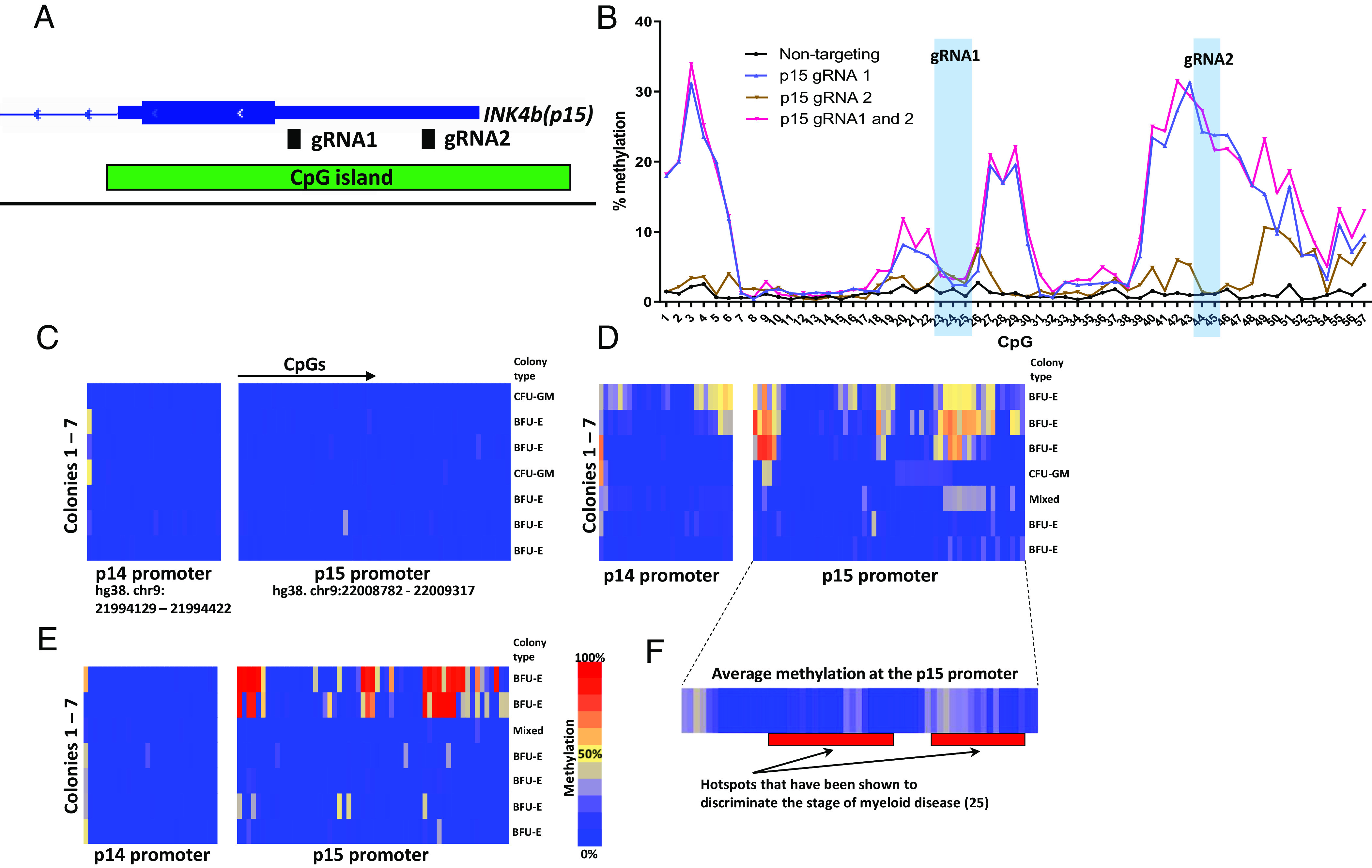
dCas9 3A3L-targeted methylation is dependent on gRNA location and hypermethylation at p14 and p15 promoters is maintained in individually picked colonies. (*A*) Location of gRNAs 1 and 2 at the p15 promoter. (*B*) Percentage DNAm per CpG across p15 after targeting with dCas9 3A3L and a nontargeting gRNA (pink), gRNA 1 (blue), gRNA 2 (green) or both gRNAs 1 and 2 (red). (*C*) Targeted bs-seq data from p14 and p15 promoters in 7 colonies 14 d after nucleofection with dCas9 3A3L-mut. Each row represents data from a single colony and each column is percentage methylation data from an individual CpG. The type of colony is shown on the *Right*. (*D*) Targeted bs-seq data from 7 colonies nucleofected with dCas9 3A3L targeting DNAm to p14, p15, and p16. (*E*) Targeted bs-seq data from 7 colonies nucleofected with dCas9 3A3L targeting DNAm to p15. (*F*) Average methylation at the p15 promoter overlapped with hotspots of CpG methylation identified in a previous study (25) that found increasing methylation from normal to diseased bone marrow.

The pattern of DNAm we observed across the p15 promoter was highly reproducible across different experiments (Fig. 1*C* and *SI Appendix*, Fig. S3 *A* and *B*). To assess whether this was dependent on the gRNA position, we designed a second gRNA for the p15 promoter (Fig. 2*A*) and targeted DNAm in CD34^+^ cells before the CFU assay. Targeting DNAm using gRNA 1 or both gRNAs 1 and 2 produced a consistent pattern of DNA hypermethylation across the p15 promoter; however, using gRNA 2 alone resulted in very little methylation (Fig. 2*B*). This was reproducible across independent biological replicates (*SI Appendix*, Fig. S4*A*), indicating that targeting different regions of the CpG island can change the efficiency of methylation deposition and spreading.

### Individual Myeloid Colonies Show Variable Levels of DNA Hypermethylation.

The CFU assay offers a unique advantage compared to liquid culture as one can isolate individual myeloid colonies that have differentiated from a single CD34^+^ cell into thousands of progenies. To assess DNAm within individual colonies, we targeted methylation using the dCas9 3A3L or 3A3L-mut mRNA and either a single gRNA directed to p15, or a pool of three gRNAs to p14, p15, and p16. We picked individual burst-forming unit-erythroid (BFU-E) colonies or CFU granulocyte–monocyte (CFU-GM) as these are the largest colonies (*SI Appendix*, Fig. S4*B*) and give robust targeted bs-seq data.

As expected, there was little CpG methylation in the control group (Fig. 2*C*). After targeting DNAm to p14, p15, and p16, we found three out of seven colonies had CpGs with up to 83% hypermethylation at the p15 promoter, and 55% hypermethylation at p14 (Fig. 2*D*). When using only the p15 gRNA and dCas9 3A3L, two colonies had methylation targeted to p15 (Fig. 2*E*). In these two successfully targeted colonies, some CpGs were up to 99% methylated (as shown by the red rectangles in the figure), showing that some hypermethylation was faithfully inherited at that CpG site in virtually all differentiated cells. We also cultured nucleofected CD34^+^ cells for 48 h and 72 h in serum-free conditions before seeding into the CFU assay and found 7 out of 10 colonies with similar levels of methylation maintenance after 14 d (*SI Appendix*, Fig. S4*C*).

### DNAm at p15 May Be Clinically Relevant.

We next wanted to know whether the pattern of hypermethylation at p15 had clinical relevance, as p15 is frequently methylated in myeloid neoplasm and AML (22–25). Previous work has quantitatively assessed p15 promoter hypermethylation in normal bone marrow compared to progressive stages of myeloid disease and found hotspots of CpG methylation, which increases as disease progresses (25). We mapped the two regions (35 CpGs in total) identified in Brekensiek et al. with our DNAm peaks after CRISPR/dCas9 targeting and found 54% (19 of 35) of the CpGs in our study had greater than 10% hypermethylation (Fig. 2*F*). Importantly, this suggests targeting DNAm using CRISPR can recapitulate disease-relevant hypermethylation events which are maintained in myeloid cells.

**Fig. 3.**
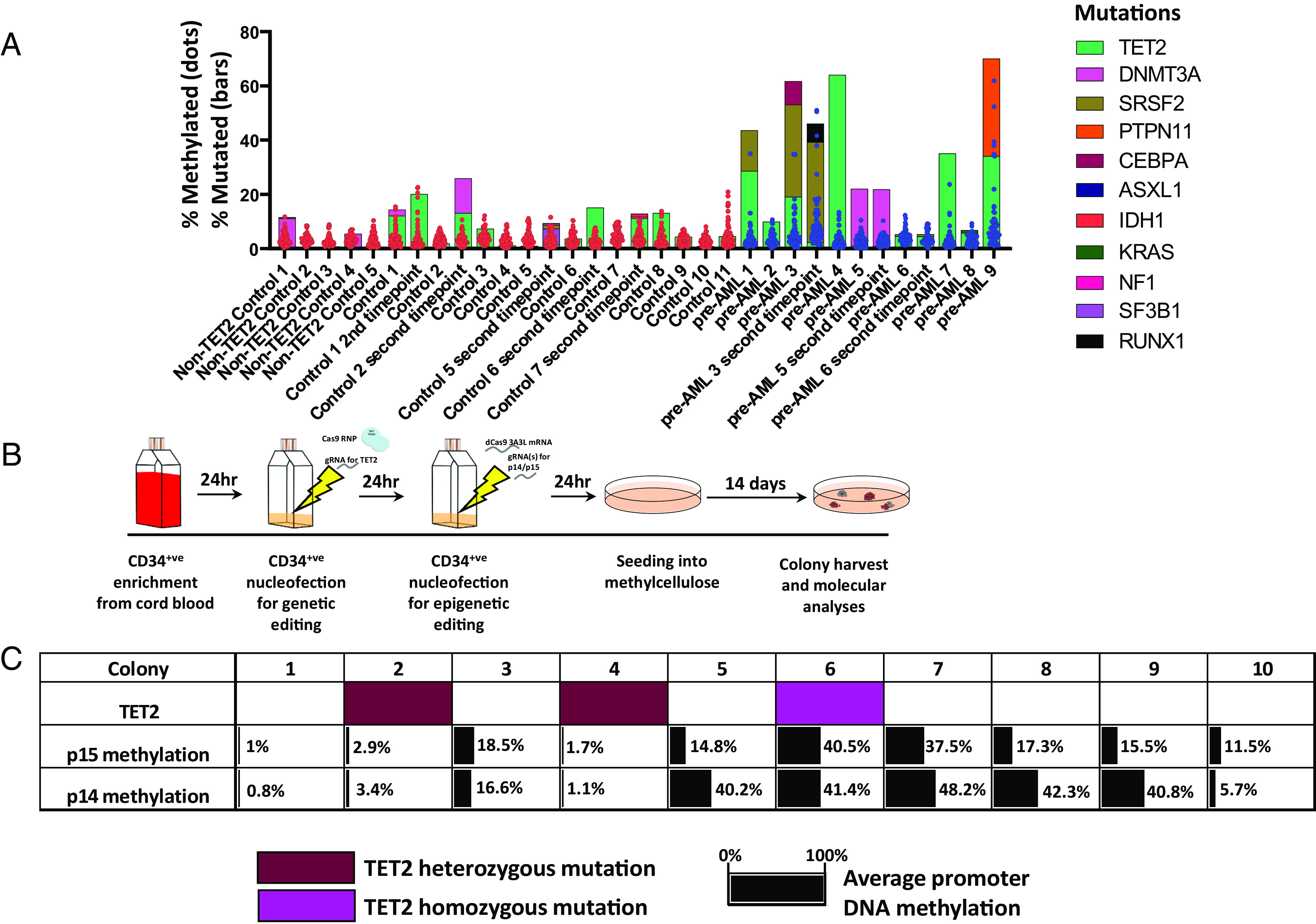
p15 promoter methylation occurs in some CHIP patients and dual genetic and epigenetic editing is possible in the same CD34^+^ cell. (*A*) Targeted bs-seq data at the p15 promoter from CHIP patients, each dot represents the methylation percent of an individual CpG in the promoter. Overlayed is the variant allele frequency data per patient as previously published (27). F test to measure equality of variances between control (n = 21) and pre-AML (n = 12) *P* < 0.0001. F = 17.5, DFn = 11, Dfd = 20. (*B*) Schematic showing the dual nucleofection protocol. CD34^+^ cells are first nucleofected with a Cas9 RNP complexed with a gRNA targeting TET2. Cells are cultured in serum-free conditions including cytokines for 24 h before a second round of nucleofection with the dCas9 3A3L mRNA and gRNAs targeting p14 and p15. Dual nucleofected CD34^+^ cells are cultured again for 24 h in serum-free conditions with cytokines before seeding 1,200 cells in methylcellulose for the CFU assay. (*C*) Summary of targeted sequencing of 10 picked colonies after TET2 editing with Cas9 and DNAm targeting to p14 and p15. *Top* row shows type of TET2 mutation detected per colony (heterozygous; purple. Homozygous; pink). *Middle* and *Bottom* rows show the average DNAm detected across the promoter at p15 (*Middle* row) and p14 (*Bottom* row).

To extend our clinical investigations, we also asked whether p15 hypermethylation is detectable in the peripheral blood of patients with clonal hematopoiesis of indeterminate potential (CHIP), with an identified known mutation. We selected samples from the European Prospective Investigation into Cancer (EPIC) cohort with known CHIP mutations in a previously published study (27). By performing targeted bs-seq on 33 CHIP samples (21 control and 12 pre-AML), we found some patients with CHIP had hypermethylation at the p15 promoter (Fig. 3*A*) but not the p14 promoter (*SI Appendix*, Fig. S5*A*). Although an unpaired *t* test with Welch’s correction showed no significant difference between the mean of the control vs. pre-AML groups, the F test to compare variances showed a significant difference; suggesting more variation in methylation at p15 in the pre-AML samples compared to control. To increase the power of this test, we analyzed a further 96 CHIP patient samples for p15 methylation (75 control and 21 pre-AML; *SI Appendix*, Fig. S5*B*), but found no significant difference in the mean methylation; however, the variance was again significantly different between groups. Taken together, these data indicate that p15 hypermethylation is detectable in the peripheral blood of some patients with CHIP, and pre-AML patients have increased variability of methylation at this promoter.

### Dual Epigenetic and Genetic Editing Is Feasible in CD34^+^ Cells.

Next, to extend the disease modeling potential of this method, we established a protocol to create two sequential hits: genetic and epigenetic editing in the same CD34^+^ cell (Fig. 3*B*). Our aim was to mutate and knock out TET2 [a commonly mutated gene in AML and myeloid malignancies (28)], and hypermethylate p15/p14 in two stages, to simulate two sequential aberrant hits. After two rounds of nucleofection, we cultured CD34^+^ cells in serum-free conditions before performing a CFU assay. After harvesting bulk colonies, we confirmed that TET2 was mutated using targeted sequencing, and all mutations were nonsynonymous (*SI Appendix*, Fig. S6*A*). We found an A base insertion was the most frequent editing event using this gRNA (35% C/CA chr4:105275217; *SI Appendix*, Fig. S6*A*; 3 biological replicates). This insertion causes loss of function of TET2 protein in HSPCs (29) (*SI Appendix*, Fig. S6*B*). Targeted bs-seq analysis showed that TET2 editing alone does not result in hypermethylation at p15 (*SI Appendix*, Fig. S6 *C*, *Upper*); and p15 was successfully methylated in the bulk double-edited colonies (*SI Appendix*, Fig. S6 *C*, *Bottom*). Using targeted sequencing, we found that leaving 24 h between Cas9 targeting TET2 and dCas9 3A3L targeting p15 resulted in a small percentage of on-target mutations at the p15 promoter, increasing the time to 48 h between the editing steps prevented this on-target, erroneous editing (*SI Appendix*, Fig. S6*D*).

We next picked individual colonies to assess whether this method resulted in mutation of TET2 and methylation at p15/p14 in the same CD34^+^ cell (*SI Appendix*, Fig. S7). We analyzed ten BFU-E colonies and found that one colony had both a homozygous TET2 mutation and over 40% average p15/p14 hypermethylation (Fig. 3*C*; colony 6); demonstrating that this method can target specific mutations and promoter hypermethylation in the same CD34^+^ cell. Colonies 3, 5, and 7 to 10 had some hypermethylation at p15 and p14 without any TET2 editing and colonies 2 and 4 had heterozygous TET2 mutations without hypermethylation (*SI Appendix*, Table S3). Colony 1 was not edited at TET2, p15 or p14 (Fig. 3*C*). These results demonstrate that it is possible to create a spectrum of (epi)genetic edits at specific targets, which can be used to increase our understanding of the interplay between different aberrant mutations and epimutations and the impact on biological function.

### p15 Hypermethylated Blood Stem Cells Can Engraft the Bone Marrow of Immunodeficient Mice and DNAm Is Maintained in All Lineages.

**Fig. 4.**
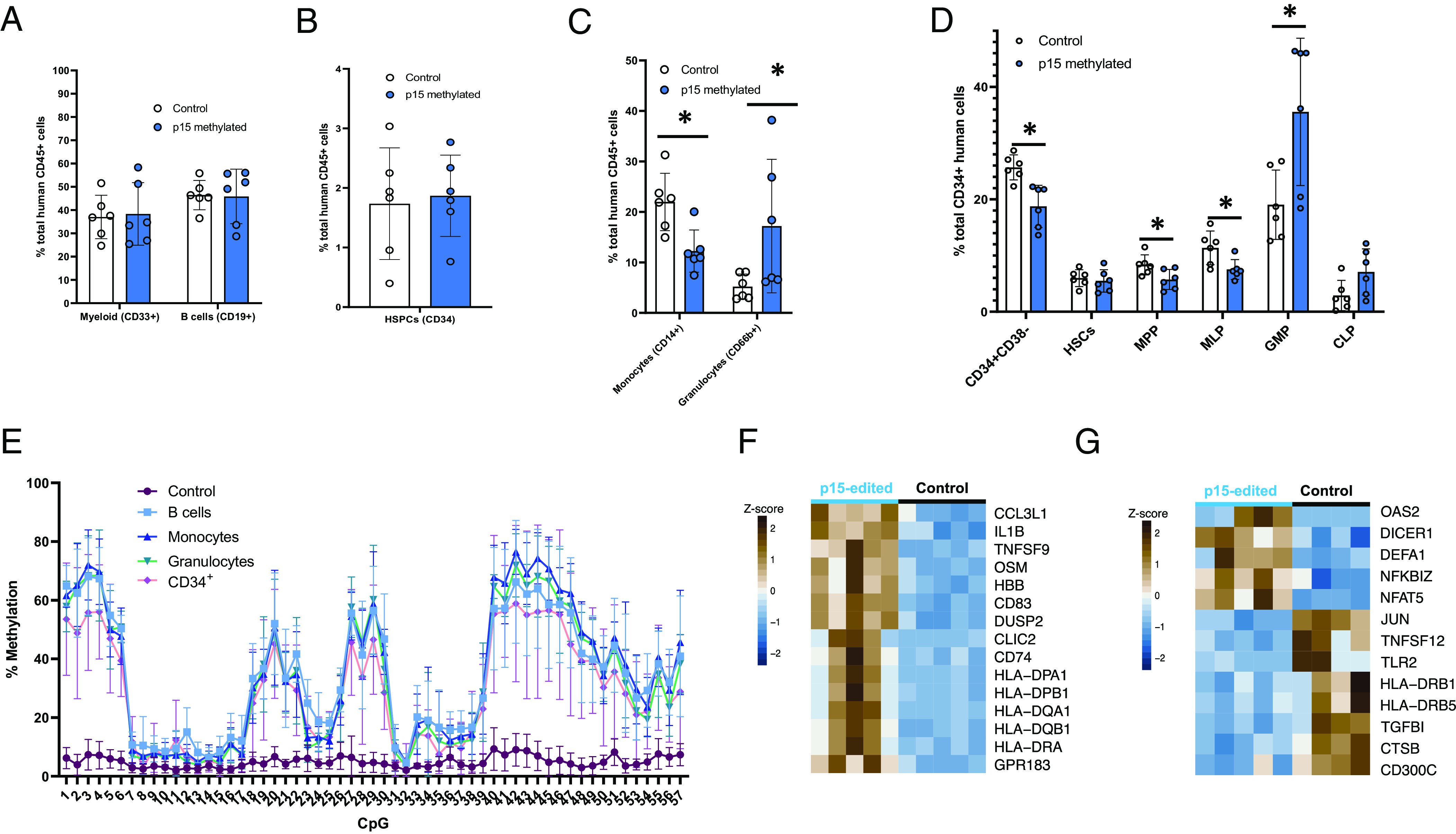
p15 methylated human HSCs can engraft the mouse bone marrow, methylation is maintained, impacts hematopoiesis, and may create an inflammatory environment. (*A*) Percentage myeloid (CD33^+^) and B cells (CD19^+^) in the bone marrow after editing with dCas9 3A3L and a nontargeting gRNA or p15 gRNA and 19 wk of engraftment. Each dot represents one humanized mouse, which was engrafted from the same pool of edited CD34^+^/CD38^−^ cells and errors bars: mean ± SEM. (*B*) Percentage CD34^+^ cells in the bone marrow after CRISPR editing and 19 wk engraftment. (*C*) Percentage monocytes (CD14^+^) and Granulocytes (CD66b^+^) cells after CRISPR editing and 19 wk engraftment. (*D*) Percentage CD34^+^/CD38^−^, HSCs (CD38^−^CD90^+^CD45RA^−^), MPP (CD38^−^CD90^−^CD45RA^−^), MLP (CD38^−^CD90^−^CD45RA^+^), GMP (CD38^+^CD45RA^+^CD135^+^CD10^−^) and CLP (CD38^+^CD45RA^+^CD10^+^) cells after CRISPR editing and 19 wk engraftment. Unpaired *t* test used for significance. *P < 0.05. (*E*) Targeted bs-seq data from the p15 promoter after CRISPR targeting and 19 wk engraftment in B cells (blue), monocytes (dark blue), granulocytes (green) and CD34^+^ cells (pink). (*F*) Significant gene expression changes in p15 methylated monocyte cells vs. control harvested from mouse bone marrow 19 wk after engraftment. (*G*) A heatmap showing up-regulated genes involved in antigen presentation and inflammatory cytokines in p15 edited granulocytes vs. control cells, 19 wk after engraftment.

Finally, to investigate the impact of p15 hypermethylation on normal hematopoiesis in vivo, we injected p15 methylated human hematopoietic stem cells (HSCs) into immunodeficient mice. We targeted methylation to p15 using dCas9 3A3L or used a nontargeting gRNA as the control in HSCs and injected them into NSG mice 48 h later. After 19 wk, the bone marrow was harvested in order to analyze the reconstitution potential and lineage output from p15-methylated HSCs. We found that p15-methylated HSCs were able to engraft (*SI Appendix*, Fig. S8*A*) and reconstitute the human hematopoietic system with no major differences in the proportions of myeloid (CD33^+^), lymphoid (CD19^+^), and progenitor (CD34^+^) cells (Fig. 4 *A* and *B*). However, within the myeloid lineage we found a significant decrease in monocytes (CD14^+^) and increase in granulocytes (CD66b^+^) output from p15-methylated HSCs (Fig. 4*C*). Furthermore, within the CD34^+^ population there were significantly fewer multipotent progenitors (MPP) and multilymphoid progenitor (MLP) cells as well as more granulocyte/monocyte progenitor (GMP) cells in the bone marrow of mice engrafted with p15-methylated HSCs (Fig. 4*D*). Strikingly, targeted bs-seq analysis showed that p15 hypermethylation is maintained in all lineages after 19 wk of engraftment, with some CpGs up to 76% methylated (Fig. 4*E* and *SI Appendix*, Fig. S8*B*) and the pattern of methylation across the p15 promoter was consistent with our in vitro data. We also seeded CD34^+^/CD38^−^ cells harvested from the bone marrow into a CFU assay and saw similar numbers of colonies (*SI Appendix*, Fig. S8*C*).

### Transcriptome Analysis Suggests p15 Hypermethylated HSCs Give Rise to Monocytes and Granulocytes with Distinct Immune Effector Functions.

Monocytes and granulocytes harvested from engrafted mice were analyzed using RNA-seq. Both cell types show dysregulated transcriptional programs related to effector immune functions when derived from p15-HSPCs. In the monocytes, a significant proportion of the genes up-regulated are involved in mediating the activation of the immune response (*SI Appendix*, Fig. S9 *A* and *B*), particularly genes involved in antigen presentation (HLA genes) and proinflammatory cytokines (IL1B, TNF) (Fig. 4*F*). Distinctively, in the granulocytes, receptors (DICER1, TLR2) and transcription factors involved in pathogen recognition and the type I interferon response (JUN, NFAT5) (30, 31) appear differentially expressed (Fig. 4*G*), suggesting a potential defect to mount balanced antiviral and inflammatory responses (*SI Appendix*, Fig. S9 *C* and *D*). Overall, the transcriptional analysis reveals that innate immune cells which have inherited p15 methylation from the HSCs in vivo could cause an exacerbated inflammatory environment.

In conclusion, we have established a method for targeting DNAm using dCas9 3A3L in human HSPCs and shown that myeloid cells maintain methylation at p14 and p15 during differentiation, repressing the p15 gene. Individual colony analysis revealed some CpGs retained up to 99% methylation, indicating that this can be a persistent change in myeloid cells. We have also demonstrated the feasibility of dual (epi)genetic editing in CD34^+^ cells, which will be useful for distinguishing the contributing role of aberrant events in initiating myeloid cancer. Finally, we have shown that p15 hypermethylated human HSPCs can reconstitute the bone marrow of mice and methylation was maintained for a least 19 wk, affecting the proportions of some progenitor cells, monocytes, and granulocytes in the mouse bone marrow as well as resulting in monocytes, which may create an inflammatory environment. This indicates that p15 hypermethylation may disrupt normal hematopoiesis over time, a finding that warrants further investigation based on our discovery of p15 methylation in CHIP patient peripheral blood.

## Discussion

Using an optimized form of CRISPR/dCas9 3A3L, we demonstrate that HSPCs can be edited to induce disease-specific DNAm signatures, and that this ectopic DNAm is maintained as cells differentiate into subsequent progeny in vitro and in vivo. Moreover, we show that hypermethylation of p15, as seen in myeloid disease, derails differentiation of HSPCs in vivo. Edited DNAm was also maintained at p14 and p15 gene promoters as myeloid progenitor cells differentiated in vitro, reducing p15 gene expression, without affecting colony-forming potential. Colonies had up to 99% methylation at some CpGs, suggesting de novo DNAm was permanently inherited during myeloid differentiation. P15 methylated stem cells can engraft the mouse bone marrow, and methylation is maintained in CD34^+^, myeloid, and lymphoid lineages (up to 76% at some CpGs). p15 methylated stem cells successfully recapitulated normal hematopoiesis; however, after 19 wk, there were differences in the proportions of monocytes and granulocytes as well as some progenitor cell fractions. This finding implies that de novo promoter hypermethylation events in HSCs can be maintained permanently and may have an impact on hematopoiesis in vivo.

The DNAm maintenance we found at p15 in vitro occurred in both the CFU assay and serum-free liquid culture. In the bone marrow of humanized mice, we showed that hypermethylated p15 is maintained in differentiated granulocytes, monocytes, and B cells, demonstrating both lymphoid and myeloid lineages inherit this DNAm from HSCs. This finding may be clinically relevant since we detected p15 hypermethylation in the peripheral blood of patients with clonal hematopoiesis, a finding in line with recent evidence of increased epigenetic age in CHIP patients (32), suggesting that p15 hypermethylation (and other epigenetic changes) may be present in human HSPCs in the bone marrow. Our in vivo data indicate that p15 methylation contributes to changes to hematopoiesis in the bone marrow after 19 wk, and the transcriptome analysis suggests that the monocytes become activated and create an inflammatory environment. It would be of potential interest to study how changes in the methylome affect HSC response to emergence hematopoiesis triggered by different environmental stress known to drive clonal hematopoiesis.

A frequent problem when targeting de novo DNAm has been the potential for off-target effects, which can be substantial (15). Surprisingly, we did not find significant off-target hypermethylation implementing a strict Bonferroni correction when comparing dCas9 3A3L + p15 gRNA to dCas9 3A3L-mut or the nontargeting control. However, we must caveat that the genome wide DNAm EPIC array analysis only examines a small, but functionally enriched, portion of the human DNA methylome (~3% of the total CpGs). Although, even current “gold standard” whole-genome bisulfite sequencing cannot evaluate the entire DNA methylome robustly, and definitely exclude off-target changes, due to both methodological limitations and genomic complexity (33–36). Therefore, while the on-target results were the most statistically rigorous, we cannot completely exclude off-target effects in our experiments beneath this threshold. To test robustly whether these exist and are experimentally reproducible or stochastic (which would fail through reproducibility tests but would still be inherited during HSPC differentiation, as our evidence shows) would require a different experimental design, more powered statistically.

It is well established that deletion or silencing of the three crucial cell cycle regulators targeted in this study, located in close proximity in the genome (p15, p16 and p14) is common in many types of cancers (37). Specifically for hematopoiesis and hematological malignancy, knocking out p16 and p14 in mice has been shown to increase HSC self-renewal and repress differentiation, while also promoting cancer (38–41); however, the impact of p15 loss is unclear as when deleted in mice, p15 does not significantly increase cancer development without additional perturbations and p15 knockout promotes HSCs differentiation (42–44). Other evidence suggests p15 expression does play a tumor-suppressive role, as deleting the entire locus containing p15, p14, and p16 exacerbates tumorigenesis in mice further than p14 and p16 double knockout (45). In our study, we wanted to examine the effect of repressing p15 via DNAm, while maintaining functional p16 in myeloid cells. This is clinically relevant as p15 is frequently hypermethylated in AML and myeloid dysplasia/neoplasm but p16 remains intact, a seemingly unique pattern of disruption at this loci to myeloid disease compared to other cancers (37).

In our study, we saw no effect in vitro after targeting p15 methylation; however, after 19 wk of engraftment, we analyzed the mouse bone marrow and found differences in hematopoiesis with changes to the number of some progenitor fractions plus decreased monocytes and increased granulocytes. Monocyte gene expression changes indicate they are “preactivated” and may contribute to an increased inflammatory environment in vivo. This indicates that the biological impact of p15 hypermethylation on HSC function and differentiation, may only be revealed as cells age in the bone marrow, a clinically relevant finding given our detection of p15 hypermethylation in the peripheral blood of patients with clonal hematopoiesis; a predominantly aging-associated phenomenon with a risk of progressing to AML (27, 46). Future work will involve deeper characterization of how cells derived from p15 hypermethylated HSC respond to exogenous stimuli and can shape the evolution of clonal hematopoiesis as it has been recently described for TET2-edited human HSCs (29). It may be that the altered gene expression patterns are established in the HSCs and inherited during differentiation or these may be established after monocytes have matured.

It is likely that extrinsic factors in the bone marrow microenvironment could influence and further exacerbate the inflammatory response from p15-hypermethylated immune cells, which parallels recent work showing that HSCs with mutations in disease-relevant genes such as TET2 or DNMT3A have enhanced survival when exposed to infection or inflammation (19, 20). Furthermore, p15 repression plays a key role in leukemic stem cell self-renewal (47). Future work to modulate the inflammatory network could reveal whether p15 methylation, in the absence of mutations, is sufficient to dysregulate hematopoiesis in vivo.

Finally, we demonstrated in vitro the technical feasibility of dual epigenetic and genetic editing in the same CD34^+^ cell using CRISPR/Cas9. Assessing how the combination of TET2 editing and p15 methylation impacts hematopoiesis in vivo would be an exciting future direction for this work. Furthermore, this method can be used to create different combinations of genetic and epigenetic edits in the same HSPC to test the functional impact on hematopoiesis, which will be a highly relevant model system in helping understand the functional impact of genetic and epigenetic changes that are being uncovered in CHIP patients.

In summary, we have adapted CRISPR/dCas9 delivery to target DNAm in HSPCs and have shown this is maintained at the p15 promoter during hematopoiesis in vitro and in vivo. Further investigation into the impact of p15 methylation in HSCs over time in vivo may reveal insights into why p15 is frequently hypermethylated in myeloid disease, unlike other cancers. By using CRISPR tools we can begin to separate the role that DNAm and gene repression plays from aberrant genetic mutations, to better understand processes that underlie cancer development.

## Methods

### Isolation and Culture of HSPCs.

Umbilical cord blood was obtained from full term donors at the Royal London Hospital (UK). The study was approved by the East London Ethical Research committee (REC: 06/Q0604/110) and all participants provided informed consent. Mononuclear cells were isolated by density centrifugation using Ficoll-Paque (GE Healthcare). For in vitro experiments CD34^+^ cells were enriched using the EasySep CD34^+^ Selection Kit II (StemCell Technologies). CD34^+^ cells were cultured in StemSpan SFEM II (StemCell Technologies) supplemented with human SCF (100 ng/µL), human FLT3 ligand (100 ng/µL) and human TPO (100 ng/µL; Peprotech) for 24 h before nucleofection using a 4D-Nucleofector system (Lonza). For in vivo engraftment experiments, we used the EasySep Human Progenitor Cell Enrichment Kit (Stem Cell Technologies) to deplete for lineage marker positive cells and HSPCs were isolated as described in detail previously (48). After sorting human HSCs (CD34^+^CD38^-^) were seeded in StemSpanSFEM (Stem Cell Technologies) supplemented with 100 ng/mL rhFLT-3L, 100 ng/mL rhSCF and 100 ng/mL rhTPO. After 48 h cells were collected for CRISPR editing.

### Samples from the EPIC.

The EPIC-Norfolk study (DOI: 10.22025/2019.10.105.00004) has received funding from the Medical Research Council (MR/N003284/1 MC-UU_12015/1 and MC_UU_00006/1) and Cancer Research UK (C864/A14136). The genetics work in the EPIC-Norfolk study was funded by the Medical Research Council (MC_PC_13048). We are grateful to all the participants who have been part of the project and to the many members of the study teams at the University of Cambridge who have enabled this research. In total, 129 samples were selected based on the highest frequency of CHIP mutations from previously published data (27).

### CRISPR/dCas9 and/or Cas9 Targeting in CD34^+^ or CD34^+^/CD38^−^ Cells.

For DNAm targeting, the dCas9 construct was fused to the catalytic domain of DNMT3A (or catalytically inactive form with C706A substitution) and portion of DNMT3L (dCas9 3A3L and dCas9 3A3L-mut) as described previously (9, 10). The efficiency of transfecting HSPCs with plasmids is low [9.3% indel frequency (49)] and causes significant cell death; whereas, nucleofection with mRNA has greater efficiency and is better tolerated [47.9% indel frequency (49)]. We cloned in the T7 promoter upstream of the dCas9 transcriptional start site and created RNA using mMESSAGE mMACHINE (ThermoFisher Scientific) following the manufacturer’s instructions. Following purification RNA was quantified using the Qubit (ThermoFisher Scientific) and concentrated to 2 µg/µL. For TET2 genetic editing, Alt-R S.p. HiFi Cas9 Nuclease V3 ribonucleoprotein (RNP) was used (Integrated DNA Technologies). Guide RNAs were designed to target p14, p15, p16, and TET2 (*SI Appendix*, Table S4) using Benchling Biology Software (https://www.benchling.com/molecular-biology/) and modified synthetic single gRNAs (sgRNAs) were ordered commercially (Synthego).

For DNAm targeting experiments, 50,000 CD34^+^ cells were nucleofected using a 4D-Nucleofector system (Lonza) in 20 µL of nucleofector solution using a nucleocuvette strip following the manufacturer’s instructions. Before nucleofection, 1.2 µg dCas9 3A3L or dCas9 3A3L-mut were combined with sgRNAs targeting p14, p15, and p16 to give a final concentration of 180 pmol of total sgRNA. After nucleofection, 80 µL StemSpan II was added to the nucleocuvette before transferring the CD34^+^ cells to a plate containing more StemSpan II media supplemented with cytokines as described above. For in vivo engraftment experiments, CD34^+^CD38^−^ human cells were electroporated using the Neon system (Thermo fisher) with 1 µg dCas9 3A3L and 150 pmol p15 gRNA or a nontargeting gRNA as described.

For dual genetic and epigenetic editing, CD34^+^ cells were combined with 6 µg Cas9 RNP (Integrated DNA Technologies), 150 pmol final concentration of sgRNA targeting TET2 and nucleofector solution to a final volume of 20 µL before nucleofection using the 4D Nucleofector. Cells were transferred to StemSpan II for 24 h before a second nucleofection with the dCas9 3A3L RNA and sgRNAs targeting p14 and p15 as described above.

### CFU Assay.

CD34^+^ cells which had undergone DNAm targeting alone or dual genetic and epigenetic editing were cultured in StemSpan II supplemented with cytokines for 24 h before seeding into the CFU assay. CD34^+^ cells were counted, and 600 cells were seeded per plate into MethoCult Classic (StemCell Technologies) after DNAm editing, or 1,200 cells per plate after dual editing. Colonies were grown in an incubator for 14 d before counting and harvesting either bulk or single colonies. For single-colony harvest, individual colonies were picked using an EVOS light microscope (ThermoFisher Scientific) and deposited directly into a PCR tube for analysis.

### Targeted Sequencing for Epigenetic and Genetic Analyses.

DNA was extracted from bulk colonies using a PureLink Genomic DNA Mini Kit (ThermoFisher Scientific), and 250 ng was used for bisulfite conversion (Zymo) and eluted in 10 µL. For single-colony analysis, DNA was extracted by digesting cells using proteinase K (ThermoFisher Scientific) at 55 °C for 30 min and RNAse A for 5 min at RT before purification using AmpureXP beads (Beckman Coulter) and eluting DNA in 15 µL of PCR grade water. Half this volume was used for bisulfite conversion.

Regions to be sequenced in the p14, p15, and p16 promoters were amplified for 38 cycles using HotStarTaq (Qiagen) with bisulfite converted DNA added to a mastermix [1× reaction buffer, 2 mM MgCl_2_, 0.025 units/µL HotStarTaq, 200 µM of each deoxynucleoside triphosphate (dNTP), 0.20 μM forward and reverse primers]. The TET2 region to be sequenced was amplified using HiFi HotStart Taq (KAPA) and 10 ng of genomic DNA added to a mastermix (1× reaction buffer, 0.025 units/µL HiFi HotStarTaq, 0.20 μM forward and reverse primers). Primer sequences are listed in *SI Appendix*, Table S2.

After the first PCR amplification, PCR products were purified using AMPureXP beads before 1 µL was taken for a second round of amplification for 10 cycles to index the samples for pooling before sequencing (*SI Appendix*, Table S2). The different genomic regions were pooled per sample and the mix was sequenced using MiSeq v2 Nano 300 (Illumina). Indexed samples were deconvoluted after sequencing, and integrity of sequencing data was checked using FastQC (v 0.11.8). Bisulfite sequencing data were aligned and analyzed using Bismark (v 0.22.1) to the human genome (hg38). For sequencing of TET2, data were aligned using bowtie2 (v 2.4.1) to the human genome (hg38), and the variant allele frequency analyzed using VarScan (v 2.4.2) with base quality >15, minimum variant allele frequency > 0.01 and *P*-value for calling variants >0.01. Data were visualized using the Integrative Genomics Viewer (50). Raw reads and data are accessible in the Gene Expression Omnibus (GEO) accession code GSE222181 (51).

### EPIC Illumina 850K Array.

The Illumina Infinium HumanMethylationEPIC (850k) array was used to analyze genome-wide DNAm differences. DNA (400 to 500 ng) was bisulfite converted and DNAm was quantified using the EPIC BeadChIP run on an Illumina iScan System using the manufacturer’s standard protocol. Raw iDAT files were processed using the ChAMP package [v.2.20.1 (52)] in R using BMIQ normalization to generate methylation β-values. The champ.DMP() function was employed to calculate differential methylated positions (DMPs) and a Bonferroni threshold of 5.768 × 10^−8^ was used to determine genome-wide significance. All downstream analysis was conducted using the GRCh37/hg19 human genome assembly. Raw reads and data are accessible in the Gene Expression Omnibus (GEO) accession code GSE222181 (51).

### RNA-Sequencing and qPCR Analysis.

RNA was extracted from samples using Direct-zol (Zymo) including an in column DNAse I treatment, before cDNA conversion (ThermoFisher Scientific). cDNA was mixed with SYBR green (BioRad) and primers (*SI Appendix*, Table S2) before qPCR to assess gene expression. Gene expression was normalized to RPS14 and data analyzed using the Pffaffl method (53).

Monocytes and granulocytes harvested from mouse bone marrow were prepared for RNA-seq using a low input Smart-seq2 protocol (library preparation and sequencing by Novogene). Reads were trimmed using Trimgalore v0.6.5 and mapped using hisat2.2.1 to the hg38/GRCh38 genome assembly. Gene counts were generated using featurecounts v2.0.3 (54). DESeq2 Bioconductor package v1.40.0 (55) was used on the RNA-Seq data to conduct differential expression analyses. *P*values were adjusted using the Benjamini–Hochberg procedure. Genes with an adjusted *P* value smaller than 0.05 and an absolute log2fold change higher than 1 were assigned as differentially expressed. For the enrichment analysis, hallmark gene sets (56) were used in a gene set enrichment analysis using clusterProfiler R package v4.8.0 (57). Raw reads and data are accessible in the Gene Expression Omnibus (GEO) accession code GSE222181 (51).

### Generation of Humanized Mice Reconstituted with CRISPR-Edited Human HSCs.

(NOD.Cg-Kit^W-41J^ Prkdc^scid^ Il2rg^tm1^) were originally obtained from The Jackson Laboratory. Mice were bred in isolators with aseptic standard operating procedures in the Biological Research Facility of The Francis Crick Institute. Once weaned, mice were kept in ventilated cages. All animal experiments were performed under the U.K Home Office project license (70/8904) in accordance with The Francis Crick Institute animal ethics committee guidance. NSG-S cKit^w41/w41^ mice aged between 8 and 12 wk were used. Forty eight hours after the CRISPR editing with dCas9 3A3L, and p15 gRNA or a nontargeting gRNA as described, cells were engrafted into mice via intravenous injection. After 19 wk, mice were killed, the bone marrow harvested and cell populations analyzed via flow cytometry (myeloid CD33^+^, B cells CD19^+^, monocytes CD14^+^, granulocytes CD66b^+^, HSPCs CD34^+^; within CD34+, HSCs CD38-CD90+CD45RA-, MPP CD38-CD90-CD45RA−, MLP CD38-CD90-CD45RA+, GMP CD38+CD45RA+CD135+CD10− and CLP CD38+CD45RA+CD10+. B cells, monocytes, granulocytes and CD34^+^ cells were isolated for targeted bs-seq analysis at the p15 promoter as described.

### Statistical Analysis.

Significance testing was performed using Prism (v 8.3.0) and *t* tests, one- or two-way ANOVA with post hoc test as specified in the figure legends. All experiments were repeated at least 3 times unless stated otherwise. Where applicable, data are plotted as mean ± SEM.

## Supplementary Material

Appendix 01 (PDF)

## Data Availability

RNA-seq and EPIC array data that support the findings of this study have been deposited in Gene Expression Omnibus (GEO) the accession code GSE222181 (51).
