# Supplementary material for: CRISPR/dCas9 DNA methylation editing is heritable during human hematopoiesis and shapes immune progeny
Source: Proc Natl Acad Sci U S A. 2023 Aug 14;120(34):e2300224120. doi: 10.1073/pnas.2300224120 (PMC10450654; doi:10.1073/pnas.2300224120)
Supplement: Supplementary file 1 — Appendix 01 (PDF) [file pnas.2300224120.sapp.pdf]

CRISPR/dCas9 DNA methylation editing is heritable during human hematopoiesis and shapes immune progeny

Emily A. Saunderson[1], Hector Huerga Encabo[2], Julie Devis[3], Kevin Rouault-Pierre[1], Marion Piganeau[2], Christopher G. Bell[4], John G. Gribben[1], Dominique Bonnet[2], Gabriella Ficz[1]
[1] Centre for Haemato-Oncology, Barts Cancer Institute, John Vane Science Centre, Charterhouse Square, Queen Mary University of London, London EC1M 6BQ, U.K.
[2] Haematopoietic Stem Cell Laboratory, Francis Crick Institute, London NW1 1AT, U.K.
[3] Group of Computational Biology and Bioinformatics, de Duve Institute, Université Catholique de Louvain, 1200 Brussels, Belgium
[4] William Harvey Research Institute, Barts and the London Faculty of Medicine and Dentistry, Queen Mary University of London, London, EC1M 6BQ, U.K.

Dr. Gabriella Ficz
Email: g.ficz@qmul.ac.uk

This PDF file includes:

Figures S1 to S9
Tables S1 to S4

Supplementary Figure 1

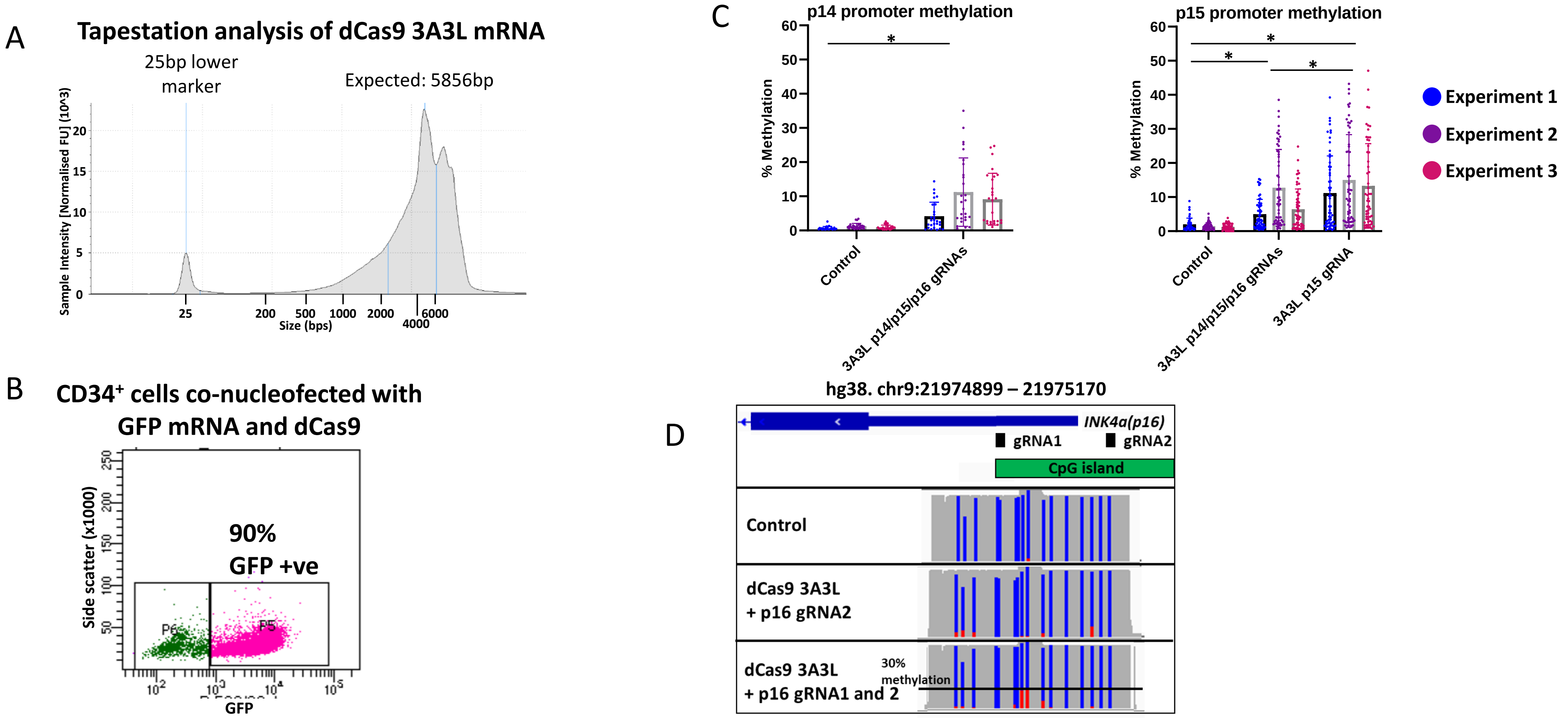

Supplementary Figure 1. A) Tapestation analysis of dCas9 3A3L mRNA produced after in vitro transcription. B) Percentage GFP^+ $CD34^+$ cells 24 hours after co-nucleofection with a cocktail of GFP mRNA, dCas9 3A3L mRNA and gRNAs targeting p14, p15 and p16. C) Three independent replicates of DNA methylation maintenance at the p14 promoter (left) and the p15 promoter (right) after dCas9 3A3L-mut or dCas9 3A3L targeting to p14, p15 and p16 or p15 alone. Each dot represents the methylation percentage of an individual CpG in the promoter. p14 promoter ($n = 3$; two-way ANOVA with repeated measures and Bonferroni's post-hoc test; *, $p < 0.05$). p15 promoter ($n = 3$; two-way ANOVA with repeated measures and Bonferroni's post-hoc test; *, $p < 0.05$). D) Targeted bs-seq data from bulk colonies harvested after CFU visualized using the Integrative Genomics Viewer. Percentage DNA methylation data from the p16 promoter is displayed with fraction of unmethylated (blue) and hypermethylated (red) shown as vertical lines; each line represents data from an individual CpG.

Supplementary Figure 2

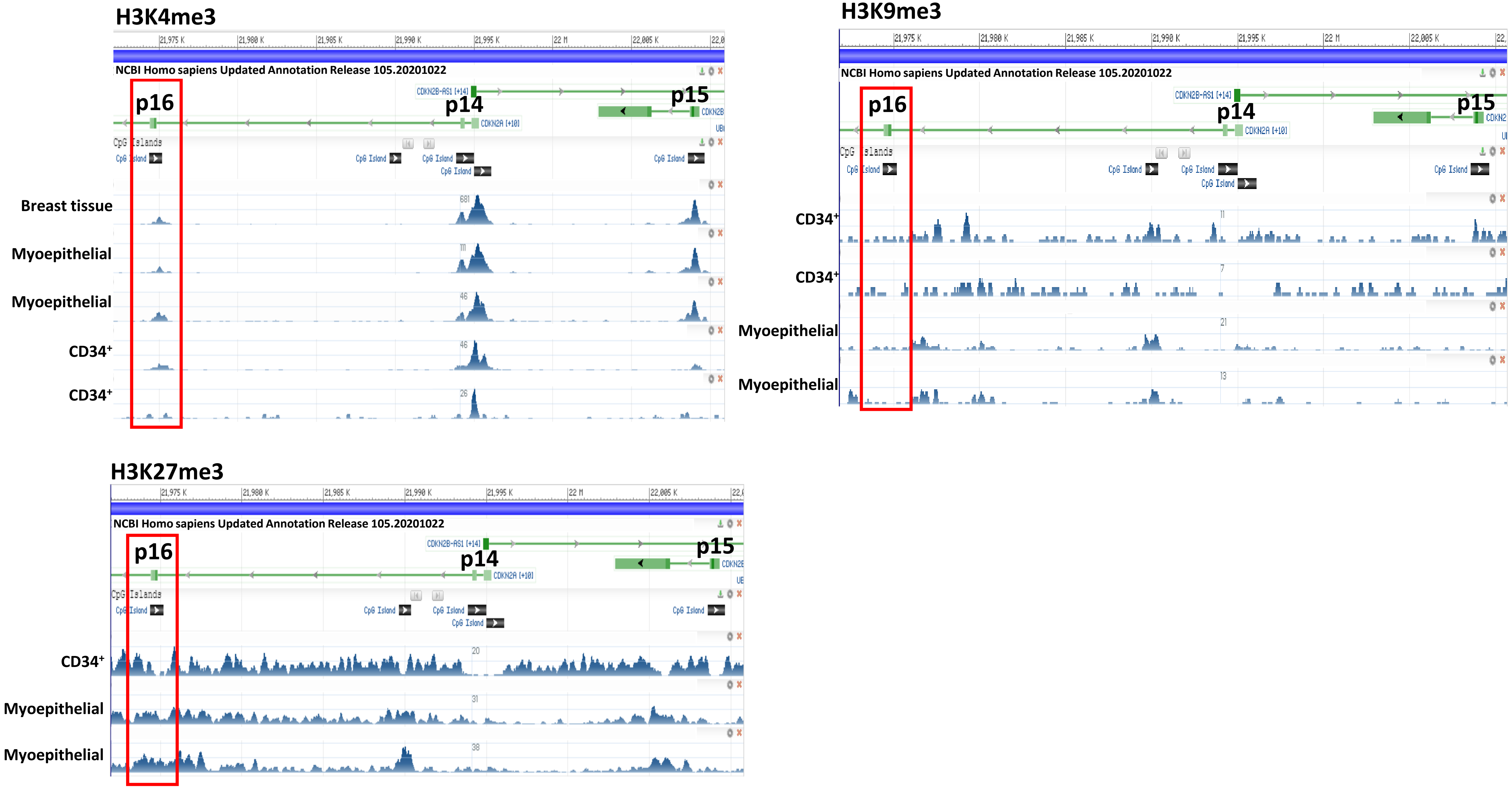

Supplementary Figure 2. Publicly available chromatin immunoprecipitation data from primary breast tissue, breast myoepithelial cells and $CD34^+$ hematopoietic stem and progenitor cells, showing peaks of H3K4me3, H3K9me3 and H3K27me3. Highlighted in red boxes is the p16 promoter.

Supplementary Figure 3

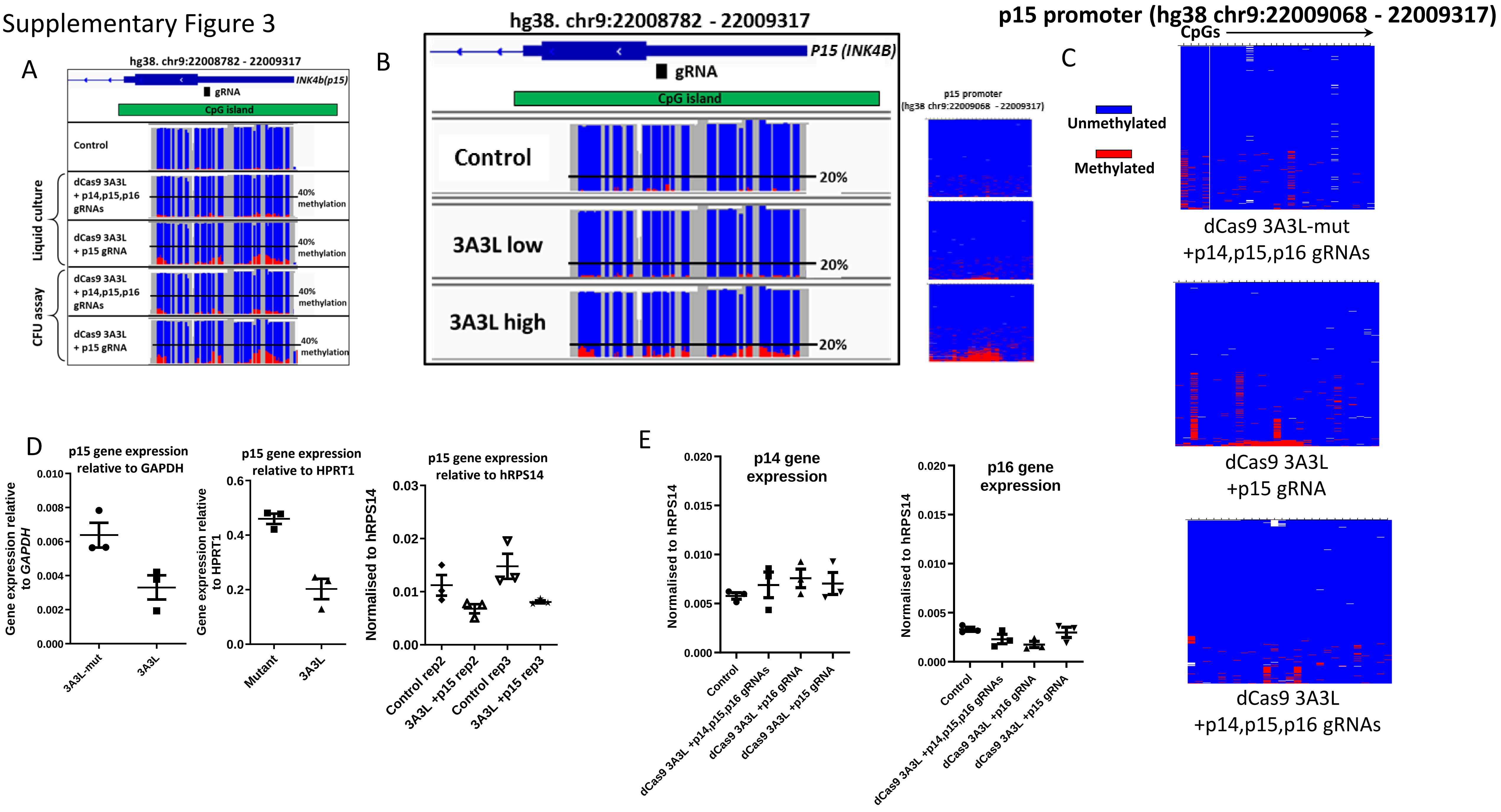

Supplementary Figure 3. A) Targeted bs-seq data from bulk colonies harvested after liquid culture (serum-free conditions containing cytokines; rows 2 and 3) or CFU assay (rows 4 and 5). $CD34^+$ cells were nucleofected with dCas9 3A3L-mut targeting p14, p15 and p16; dCas9 3A3L targeting p14, p15 and 16 or dCas9 3A3L targeting p15 alone. Percentage DNA methylation data is displayed with fraction of unmethylated (blue) and hypermethylated (red) shown as vertical lines; each line represents data from an individual CpG. B) Targeted bs-seq data at the p15 promoter from bulk colonies harvested after CFU. $CD34^+$ cells were nucleofected with dCas9 3A3L-mut targeting p14, p15 and p16 (Control); dCas9 3A3L targeting p14, p15 and p16 with lower total picomols (3A3L low) or higher total picomols (3A3L high). Data at the p15 promoter is also visualized as individual DNA strands (right panels). C) Targeted bs-seq data visualized as individual DNA strands (rows) and CpGs (columns) at the p15 promoter after targeting dCas9 3A3L-mut to p14, p15 and p16 (top); dCas9 3A3L to p15 alone (middle) or dCas9 3A3L to p14, p15 and p16 (bottom). Nucleofected cells were harvested after 48 hours in serum-free culture containing cytokines (SCF, Flt3-L and TPO) before DNA methylation analysis. D) Gene expression of p15 in bulk colonies after methylation targeting and 14 days in a CFU assay. qPCR data is normalized to GAPDH (left), HPRT1 (middle) and hRPS14 (right) expression (n = 3). E) Gene expression of p14 (left) and p16 (right) in bulk colonies after methylation targeting and 14 days in a CFU assay. qPCR data is normalized to RPS14 expression (n = 3; one-way ANOVA ns.).

Supplementary Figure 4

A

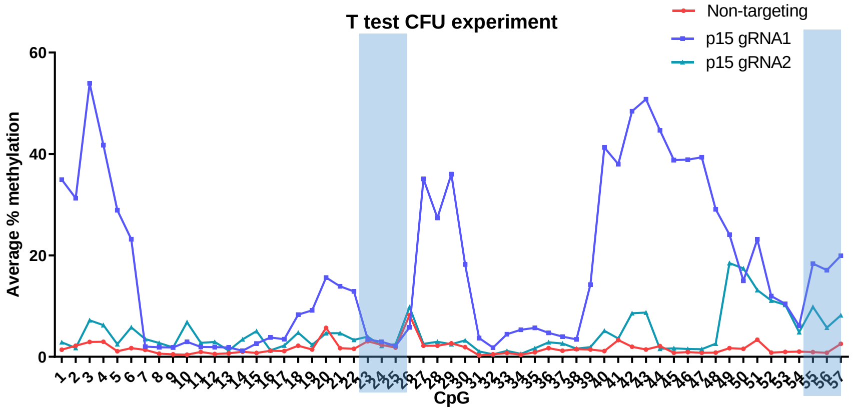

B

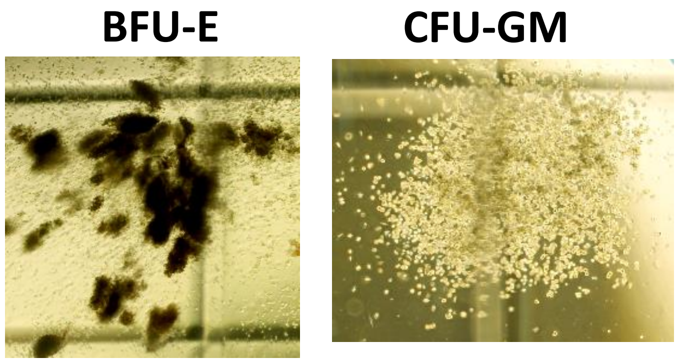

C

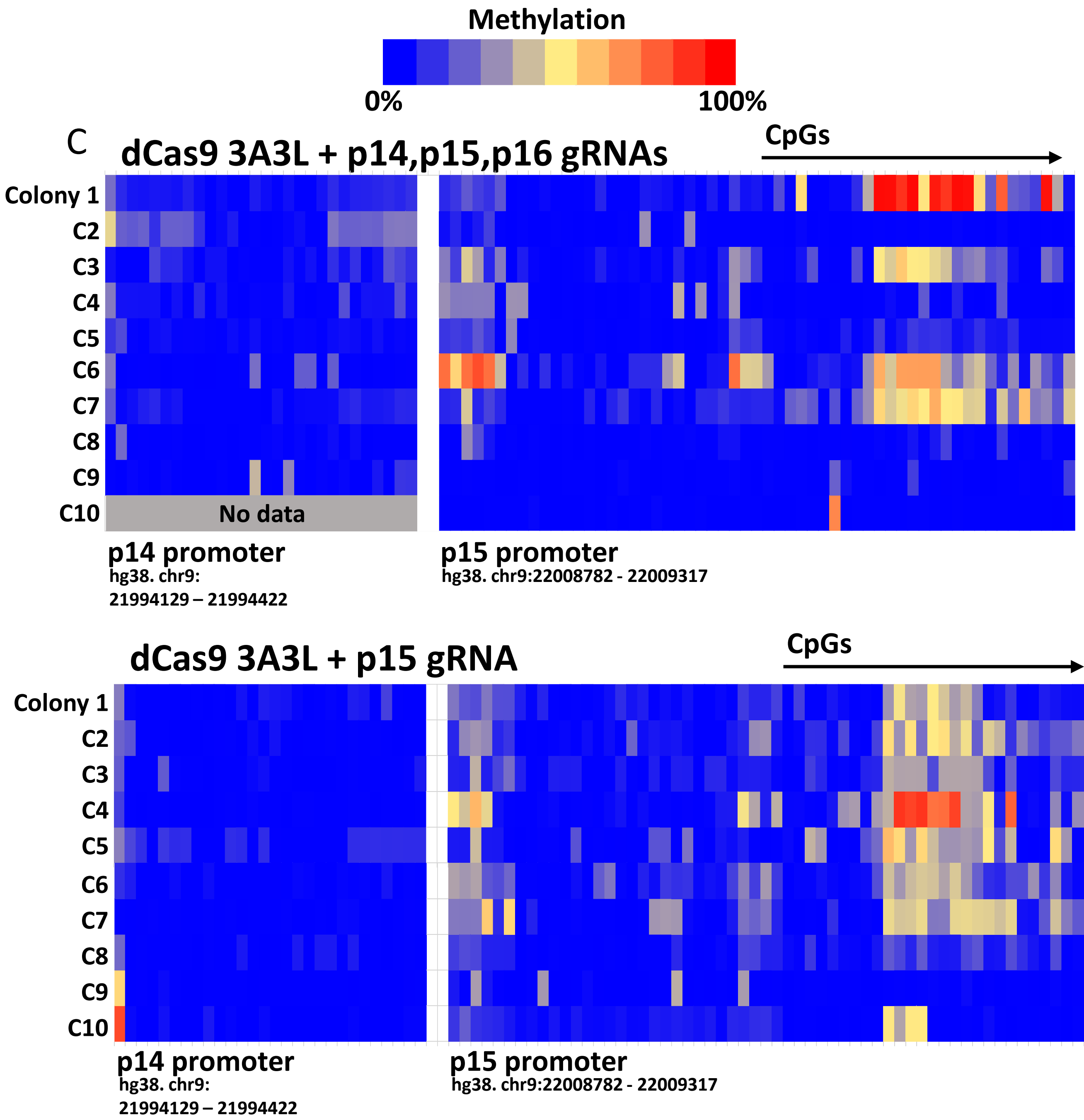

Supplementary Figure 4. A) Biological replicate experiment using gRNAs 1 and 2 to target DNA methylation to the p15 promoter. B) Representative images of BFU-E colony (left) and CFU-GM colony (right). C) Targeted bs-seq data from p14 and p15 promoters in 10 colonies 14 days after nucleofection with dCas9 3A3L targeting p14, p15 and p16 (top panel) and dCas9 3A3L targeting p15 alone (bottom panel). Each row represents data from a single colony and each column is percentage methylation data from an individual CpG. The colony number is shown on the left. $CD34^+$ cells were nucleofected and then cultured in serum-free conditions (including cytokines) for 48 hours before the 14 days CFU (colonies 1 – 5), or 72 hours before the 14 days CFU (colonies 6 – 10).

Supplementary Figure 5

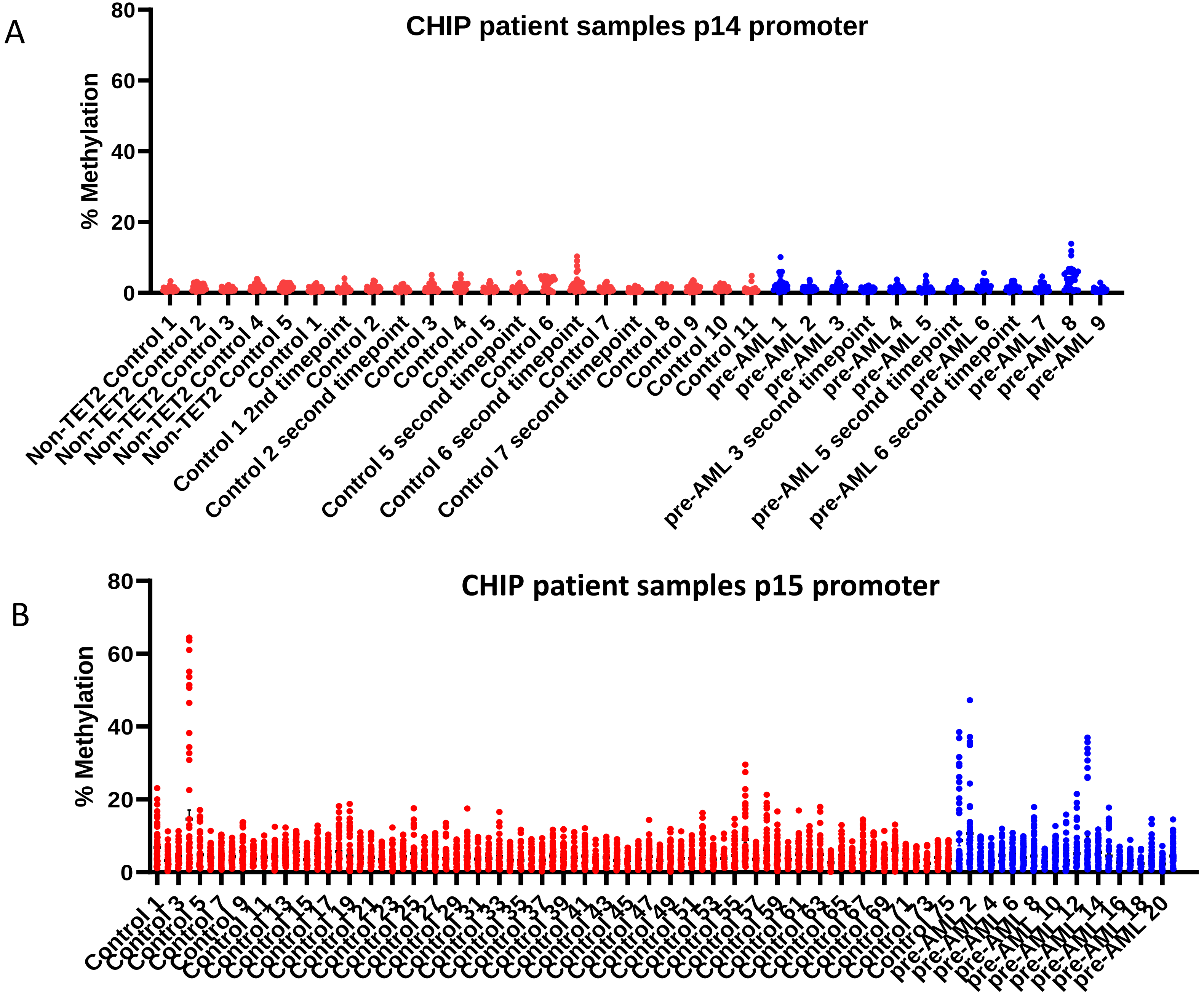

Supplementary Figure 5. A) Targeted bs-seq data from CHIP patient samples measuring DNA methylation at the p14 promoter. Each individual dot represents a single CpG from that patient. Control samples are patients with CHIP but do not develop AML (red); pre-AML patients have CHIP and develop AML during the course of the study (blue). B) 96 additional CHIP samples analyzed for p15 promoter DNA methylation using targeted bs-seq. Control samples are patients with CHIP but do not develop AML (red); pre-AML patients have CHIP and develop AML during the course of the study (blue).

Supplementary Figure 6

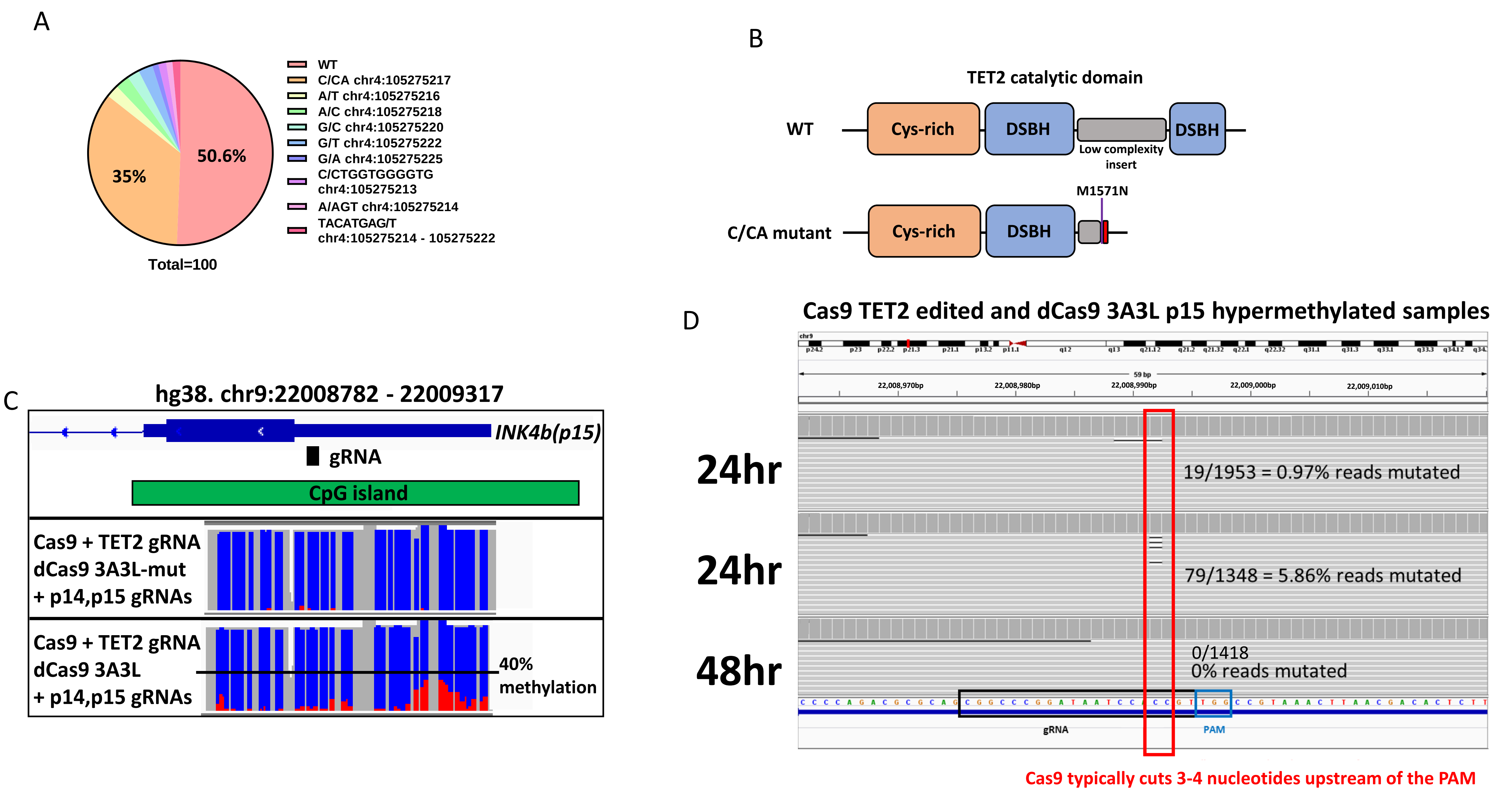

Supplementary Figure 6. A) Summary of targeted sequencing data of the region edited using Cas9 in the TET2 gene. $CD34^+$ cells were nucleofected according to the dual editing method before seeding into a 14 days CFU assay. Bulk colonies were harvested and targeted sequencing was performed at the TET2 region edited using Cas9. Mutations were detected using Varscan (v 2.4.2). B) The effect on the amino acid sequence of the TET2 protein after insertion of an A base at chr4:105275217 was assessed using an online tool (https://web.expasy.org/translate/). C) Targeted bs-seq data at the p15 promoter from bulk colonies harvested after CFU. $CD34^+$ cells were nucleofected according to the dual editing method before seeding into a 14 days CFU assay. Bulk colonies were harvested and bs-seq was performed. In the second nucleofection step, $CD34^+$ cells were targeted with dCas9 3A3L-mut targeting p14 and p15 (top panel); or dCas9 3A3L targeting p14 and p15 (bottom panel). Percentage DNA methylation data from the p15 promoter is displayed with fraction of unmethylated (blue) and hypermethylated (red) shown as vertical lines; each line represents data from an individual CpG. D) Analysis of on-target p15 promoter mutation caused by undegraded active Cas9, leaving 24 hours between genetic and epigenetic editing (top two panels), and 48 hours (bottom panel).

Supplementary Figure 7

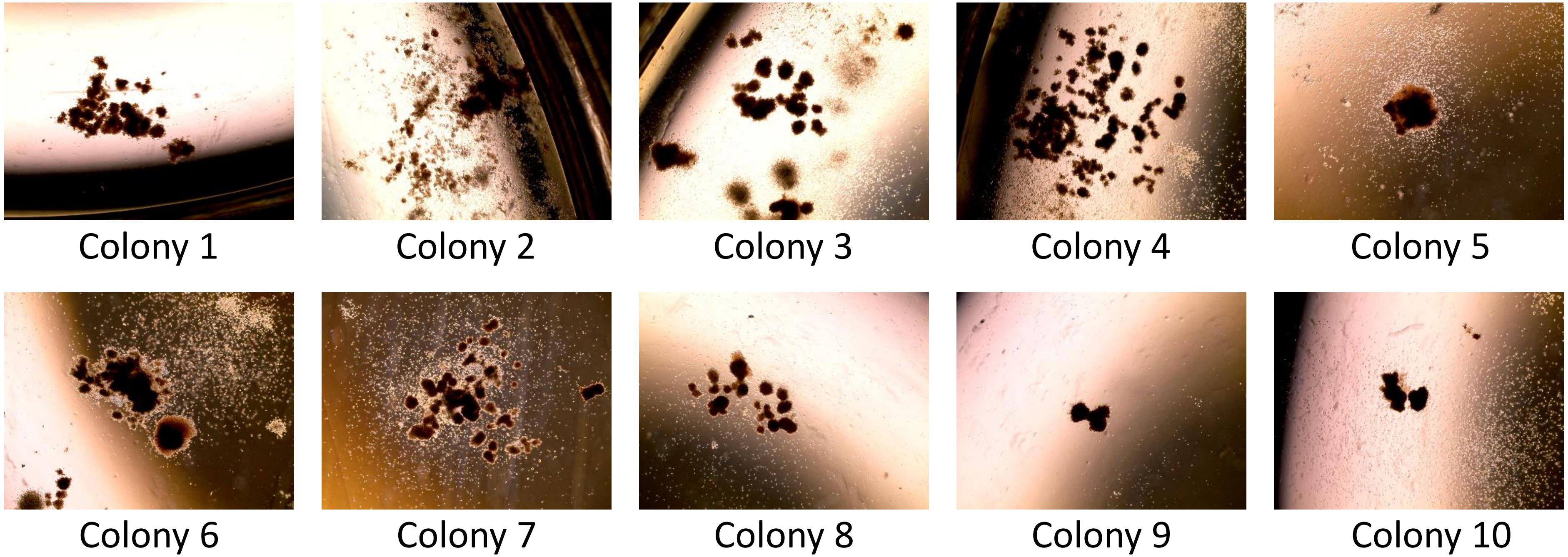

Supplementary Figure 7. CD34$^+$ cells were nucleofected with Cas9 RNP targeting TET2 and cultured for 48 hours in serum-free conditions with cytokines before a second round of nucleofection using dCas9 3A3L mRNA and gRNAs targeting p14 and p15. 24 hours after the second round of nucleofection, 1200 cells were seeded per dish into methylcellulose for a CFU assay. After 14 days 10 individual BFU-E colonies were picked as shown.

Supplementary Figure 8

A

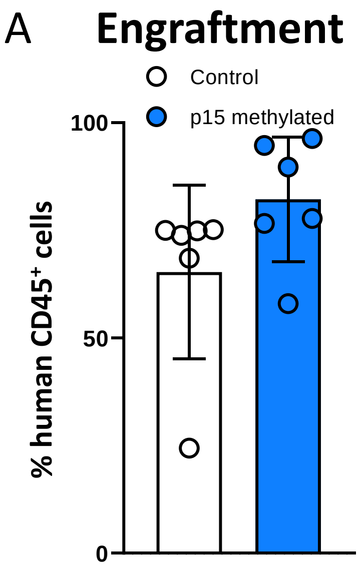

B

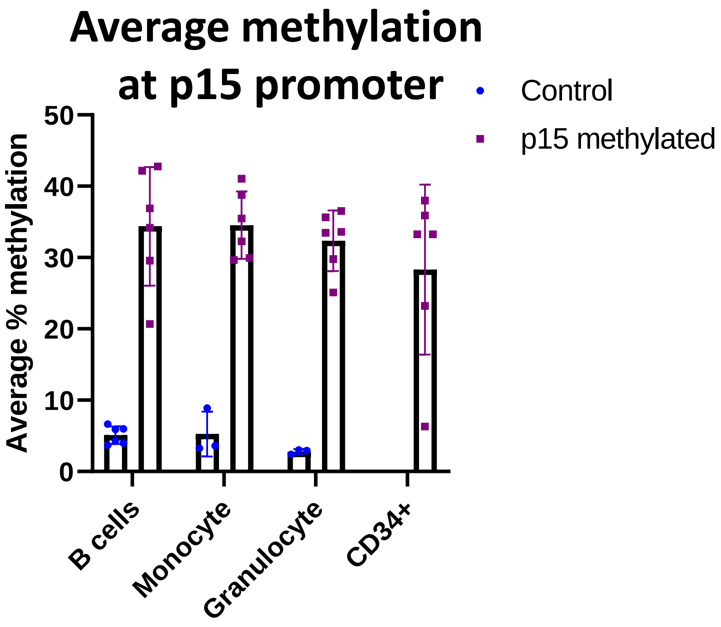

C

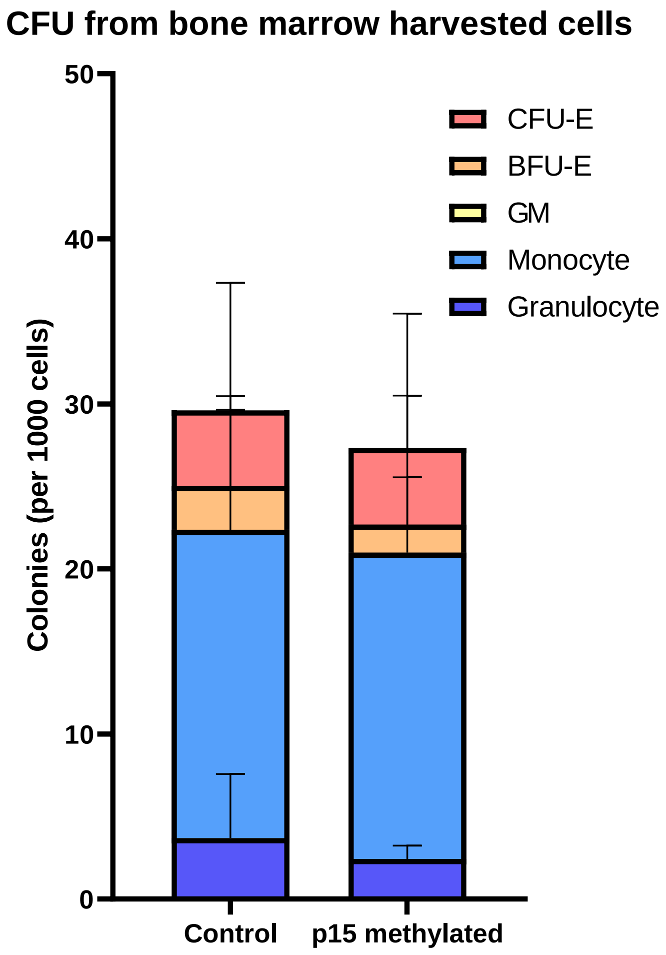

Supplementary Figure 8. A) Engraftment of human CD45$^+$ cells after CRISPR editing and engraftment in NSG-cKit$^{w41/w41}$ (NOD.Cg-Kit^{W-41J} Prkdc^{scid} Il2rg^{tm1}) mice for 19 weeks. Each dot represents one humanized mouse and errors bars: mean ± SEM. Unpaired t test used for significance, * $p<0.05$. Non-significance: NS > 0.05. B) Average DNA methylation at the p15 promoter after 19 weeks in B cells, monocytes, granulocytes and CD34$^+$. C) CFU after harvesting human HSPCs from the bone marrow of mice.

Supplementary Figure 9

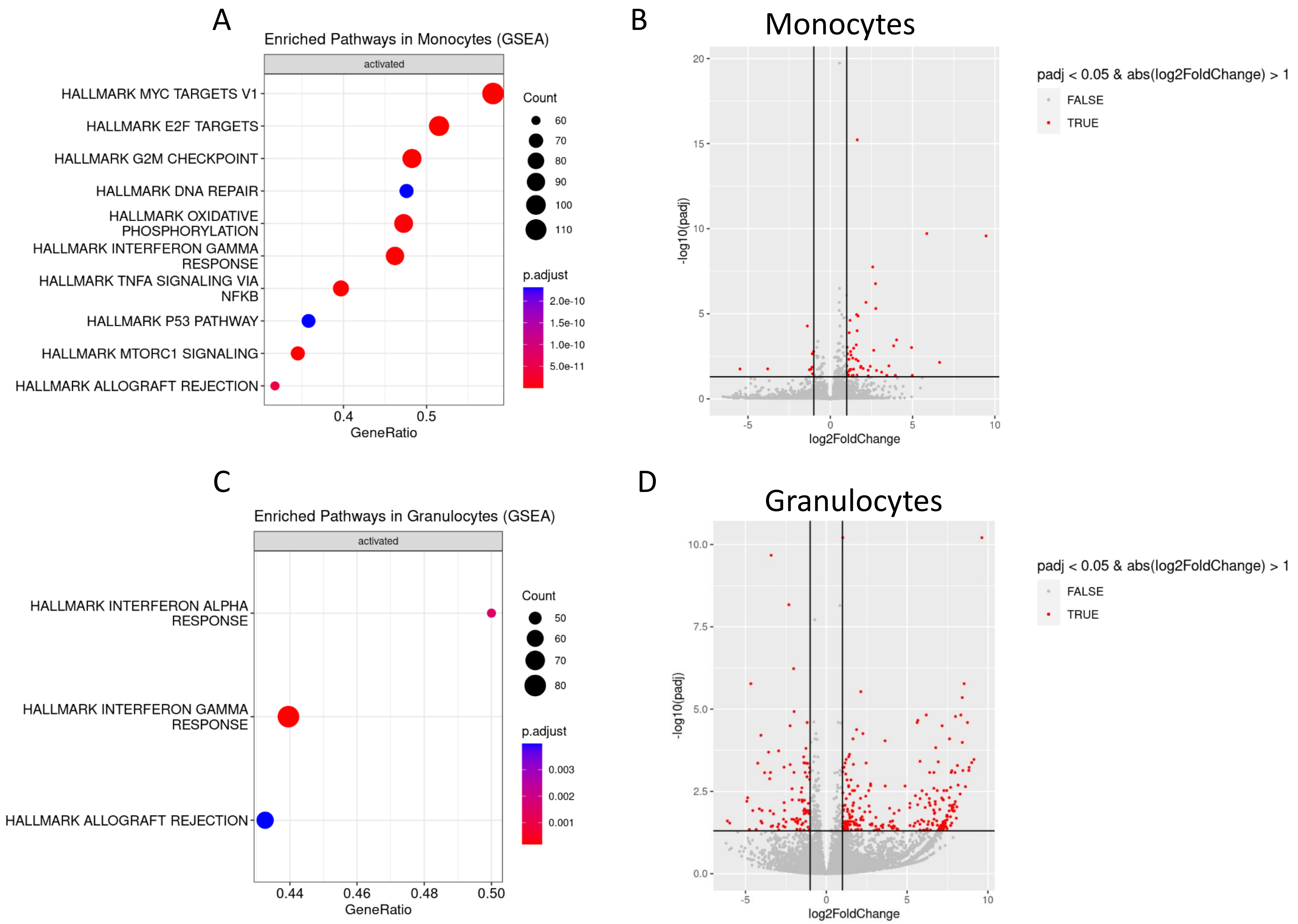

Supplementary Figure 9. A) Enriched pathways from RNA-seq data, upregulated in p15 methylated monocytes harvested from the mouse bone marrow 19 weeks after engraftment. B) Volcano plot showing significant gene expression changes in p15 methylated monocytes vs. control.C) Enriched pathways from RNA-seq data, upregulated in p15 methylated granulocytes harvested from the mouse bone marrow 19 weeks after engraftment. D) Volcano plot showing significant gene expression changes in p15 methylated granulocytes vs. control.

Supplementary Table 1

	P.Value	adj.P.Val	3a3l_AVG	mut_AVG	deltaBeta	CHR	MAPINFO	Strand	Type	gene	feature	cgi	feat.cgi	UCSC_Islands_Name	logFC	AveExpr	t
cg00525580	2.55E-09	* 0.001857	0.357523	0.01778	0.339743	9	22009352	R	II	CDKN2B	TSS200	island	TSS200-island	chr9:22008657-22009471	-0.33974	0.187651	-52.3525
cg08700949	5.54E-09	* 0.002016	0.408399	0.039166	0.369233	9	22009493	R	II	CDKN2B	TSS200	shore	TSS200-shore	chr9:22008657-22009471	-0.36923	0.223783	-46.0848
cg01716061	2.96E-08	* 0.007183	0.285239	0.040636	0.244603	9	22009518	F	II	CDKN2B	TSS1500	shore	TSS1500-shore	chr9:22008657-22009471	-0.2446	0.162938	-34.9691
cg13788469	7.34E-08	0.013354	0.268993	0.070179	0.198814	10	74124335	R	II		IGR	opensea	IGR-opensea		-0.19881	0.169586	-30.1056
cg19233179	1.34E-07	0.019483	0.27432	0.032799	0.241521	9	22009470	R	I	CDKN2B	TSS200	island	TSS200-island	chr9:22008657-22009471	-0.24152	0.15356	-27.2607
cg09677820	1.71E-07	0.020736	0.350963	0.148155	0.202808	4	1.09E+08	F	II	SGMS2	3'UTR	opensea	3'UTR-opensea		-0.20281	0.249559	-26.1799
cg13420744	2.80E-07	0.026088	0.261605	0.071455	0.19015	1	12679306	R	II	DHRS3	TSS1500	shore	TSS1500-shore	chr1:12676983-12678642	-0.19015	0.16653	-24.1336
cg00906581	2.87E-07	0.026088	0.191935	0.022806	0.169128	15	94831047	R	II		IGR	opensea	IGR-opensea		-0.16913	0.10737	-24.0316
cg23693200	3.60E-07	0.029085	0.442947	0.039162	0.403785	20	4701448	F	II	PRND	TSS1500	opensea	TSS1500-opensea		-0.40379	0.241054	-23.1463
cg02400248	6.23E-07	0.045332	0.296186	0.027955	0.268231	9	22009355	R	II	CDKN2B	TSS200	island	TSS200-island	chr9:22008657-22009471	-0.26823	0.16207	-21.1305
cg11016299	6.99E-07	0.046222	0.232923	0.068382	0.16454	1	1.13E+08	F	II	PPM1J	Body	shore	Body-shore	chr1:113256885-113258149	-0.16454	0.150653	-20.7315

Supplementary Table 1. Significantly differentially methylated probes from 850K array comparing dCas9 mut-3A3L + p15 gRNA with dCas9 3A3L + p15 gRNA. Identified using the ChAMP package (v. 2.20.1) in R using Benjamini-Hochberg post-hoc test. Significant DMPs after Bonferroni correction are identified with *.

Supplementary Table 2

	P.Value	adj.P.Val	Non_AVG	3a3l_AVG	deltaBeta	CHR	MAPINFO	Strand	Type	gene	feature	cgi	feat.cgi	UCSC_Islands_Name	logFC	AveExpr	t
cg00525580	1.46E-09	* 0.001064	0.017266	0.357523	0.340257	9	22009352	R	II	CDKN2B	TSS200	island	TSS200-island	chr9:22008657-22009471	-0.34026	0.187395	-56.5246
cg08700949	5.35E-09	* 0.001948	0.048526	0.408399	0.359874	9	22009493	R	II	CDKN2B	TSS200	shore	TSS200-shore	chr9:22008657-22009471	-0.35987	0.228462	-45.7111
cg01716061	1.37E-08	* 0.003334	0.043267	0.285239	0.241972	9	22009518	F	II	CDKN2B	TSS1500	shore	TSS1500-shore	chr9:22008657-22009471	-0.24197	0.164253	-39.1723
cg19233179	1.17E-07	0.021218	0.020532	0.27432	0.253789	9	22009470	R	I	CDKN2B	TSS200	island	TSS200-island	chr9:22008657-22009471	-0.25379	0.147426	-27.5821
cg03079681	2.73E-07	0.039739	0.029211	0.274145	0.244935	9	21994223	F	I	CDKN2A	1stExon	island	1stExon-island	chr9:21994101-21995910	-0.24493	0.151678	-23.9793
cg02400248	3.87E-07	0.046872	0.021869	0.296186	0.274317	9	22009355	R	II	CDKN2B	TSS200	island	TSS200-island	chr9:22008657-22009471	-0.27432	0.159027	-22.6446

Supplementary Table 2. Significantly differentially methylated probes from the 850K array comparing dCas9 3A3L + non-targeting gRNA with dCas9 3A3L + p15 gRNA. Identified using the ChAMP package (v. 2.20.1) in R using Benjamini-Hochberg post-hoc test. Significant DMPs after Bonferroni correction are identified with *.

Supplementary Table 3

Colony 2		
**Mutation**	**Frequency**	**Location**
A/AGT	31.13%	chr4:105275214
A/AG	30.46%	chr4:105275216
C/T	32.23%	chr4:105275217
A/C	30.20%	chr4:105275218
CATACAT/C	16.52%	chr4:105275213-105275219

Single allele (rows A/AGT, A/AG, C/T, A/C)

Allele from contaminating cells (row CATACAT/C)

Colony 4		
**Mutation**	**Frequency**	**Location**
C/A	39.35%	chr4:105275217

Colony 6		
Mutation	Frequency	Location
C/CA	96.40%	chr4:105275217

Supplementary Table 3. Summary of targeted sequencing data from the TET2 region edited with Cas9 RNP from colonies which were dual edited with Cas9 RNP targeting TET2 and dCas9 3A3L targeting p14 and p15. The mutation data from individual colonies 2, 4 and 6 are shown. Mutation frequency was assessed using Varscan (v 2.4.2).

Supplementary Table 4

gRNAs	Sequence (5' - 3')
p14	TCAGAGCCGTTCCGAGATCT
p15	CGGCCCGGATAATCCACCGT
p16	CGCCAGAGCCAGCGTTGGCA
TET2	GGATTGGGCCGTCTCATGTA
**Bisulfite targeted sequencing primers**	
p14 forward	CTACACGACGCTCTTCCGATCT GTTGTTTATTTTTGGTGTTAAAGGG
p14 reverse	CAGACGTGTGCTCTTCCGATCTCCTTTCCTACCTAATCTTCTAAAAAAC
p15 forward 1	CTACACGACGCTCTTCCGATCTGGTTGGTTTTTTATTTTGTTAGAG
p15 reverse 1	CAGACGTGTGCTCTTCCGATCTCCTAAACTCAACTTCATTACCCT
p15 forward 2	CTACACGACGCTCTTCCGATCTAGGGTAATGAAGTTGAGTTTAGG
p15 reverse 2	CAGACGTGTGCTCTTCCGATCTCTAAAACCCCAACTACCTAAATC
**Targeted sequencing primers**	
TET2 forward	CTACACGACGCTCTTCCGATCTGCCACATCACCCTCAGACAG
TET2 reverse	CAGACGTGTGCTCTTCCGATCTCATAGGGCTGGTGCTTCCAT
sequencing adapter forward primer	AATGATACGGCGACCACCGAGATCTACACTCTTTCCCTACACGACGCTCTTCCGATCT
sequencing adapter reverse primer	CAAGCAGAAGACGGCATACGAGATXXXXXXXXGTGACTGGAGTTCAGACGTGTGCTCTTCCGATCT
**cDNA primers for qPCR**	
p14 forward	CAGCCGCTTCCTAGAAGACC
p14 reverse	ACGGGTCGGGTGAGAGTG
p15 forward	GGGGACTAGTGGAGAAGGTG
p15 reverse	CTGCCCATCATCATGACCTG
p16 forward	CAACGCACCGAATAGTTACGG
p16 reverse	ACGGGTCGGGTGAGAGTG

Supplementary Table 4. Sequences of gRNAs and primers.
